# *In Silico* Target Discovery of Kaempferol: Therapeutic Effect of Kaempferol on Atopic Dermatitis through Regulation of Aryl Hydrocarbon Receptor

**DOI:** 10.34133/bmr.0270

**Published:** 2025-11-11

**Authors:** Eun-Nam Kim, Hyun-Su Lee, Nguyen Minh Trang, Jin Tae Hong, Gil-Saeng Jeong

**Affiliations:** ^1^ College of Pharmacy, Chungnam National University, Daejeon 34134, Republic of Korea.; ^2^ Department of Physiology, Daegu Catholic University School of Medicine, Daegu 42472, Republic of Korea.; ^3^ College of Pharmacy & Medical Research Center, Chungbuk National University, Cheongju 28160, Republic of Korea.

## Abstract

The aryl hydrocarbon receptor (AhR) is known to bind several exogenous ligands such as natural plant flavonoids, synthetic polycyclic aromatic hydrocarbons, and dioxin-like compounds, but its association with kaempferol (KF) is unknown. Therefore, in this study, AhR was explored as an atopic dermatitis (AD)-regulating target of KF for AD regulation using *in silico* prediction, and pharmacological prediction and various AD-induced models confirmed that KF exhibited pharmacological activity through AhR regulation. Also, the results of the study showed that KF regulated epidermal differentiation terminal proteins through the AhR pathway in HaCaT cells stimulated with TNF-α/IFN-γ, and the therapeutic effect of KF was also proven in an AD-induced mouse model and a reconstructed human skin model. In this study, AhR was explored as a KF–AD combined treatment target through *in silico* prediction analysis, and KF was proven to have an AD treatment effect through AhR regulation *in vitro* and *vivo* and in the reconstructed human skin model. In particular, KF can be used as a potent inducer of AhR signaling because it protects against AD by enhancing epidermal terminal differentiation through the AhR-mediated pathway in keratinocytes.

## Introduction

Atopic dermatitis (AD) is a multifactorial disease that is becoming increasingly common in developed countries, especially in children [1]. Its pathogenesis involves complex interactions between immune cells and keratinocytes [2]. When allergens penetrate the skin, Langerhans cells capture and present them to naive T cells, which are then primed to differentiate into T helper type 2 (Th2) effector cells under the influence of cytokines such as thymic stromal lymphopoietin (TSLP) produced by activated keratinocytes [3]. These effector T cells subsequently release interleukin (IL)-4, IL-5, and IL-13, promoting allergic inflammation. Keratinocytes, which constitute the skin’s outermost layer, play a central role in forming the epidermal barrier and protecting against exogenous pathogens [4]. Epidermal homeostasis is maintained through a continuous process of terminal differentiation [5,6], and defects in this process have been linked to increased systemic allergic responses, airway hyperresponsiveness, and elevated immunoglobulin E (IgE) levels [2,7]. Therefore, promoting proper epidermal differentiation has emerged as a therapeutic approach for AD and other inflammatory skin disorders.

Recent studies have highlighted the therapeutic potential of kaempferol (KF), a plant-derived flavonoid, in AD models [8]. However, its specific molecular targets and mechanisms remain incompletely understood. With the rise of systems-based drug discovery platforms such as network pharmacology and molecular docking, it is now possible to efficiently predict active compounds, target proteins, and mechanistic pathways, markedly reducing time and cost in early-stage research [9,10]. These tools are especially valuable in natural product research, where large databases support the prediction of pharmacological activities and target interactions, with growing interest in flavonoid compounds due to their diverse bioactivities [11,12].

The aryl hydrocarbon receptor (AhR) is a ligand-activated transcription factor that senses xenobiotics, phytochemicals, and dioxins and regulates various genes, including those in the cytochrome P450 family [13,14]. Upon ligand binding, AhR translocates to the nucleus and dimerizes with the aryl hydrocarbon receptor nuclear translocator (ARNT), subsequently inducing the expression of target genes such as Cyp1a1 and Cyp1b1 [15]. The AhR signaling pathway has been identified as a critical regulator of skin homeostasis, including antioxidative responses, anti-inflammatory effects, and terminal differentiation of keratinocytes [16–19]. It also influences the expression of key epidermal differentiation markers such as filaggrin and involucrin [20,21], which are essential for maintaining the skin barrier function [22–24]. Although some AhR ligands such as coal tar and soybean tar glitter are used clinically to treat AD and psoriasis through the activation of both the AhR and nuclear factor erythroid 2-related factor 2 (Nrf2) pathways [20], there are limited phytochemicals known to act directly on AhR for therapeutic benefit. KF (3,4′,5,7-tetrahydroxyflavone), found in a variety of fruits, vegetables, and medicinal herbs [25], exhibits anti-inflammatory, antioxidant, and antimicrobial properties [8,26–28]. While gene expression profiling such as complementary DNA microarray analysis has shown changes after KF treatment in keratinocytes, direct evidence for AhR involvement in mediating its effects against AD remains scarce.

In this study, we investigated whether KF exerts its anti-AD effects by activating AhR signaling in keratinocytes, thereby enhancing epidermal terminal differentiation and suppressing inflammation. Using database-driven pharmacological prediction, molecular docking, and both *in vitro* and *in vivo* models, we aimed to identify the key targets and mechanisms of KF in modulating AD pathogenesis.

## Materials and Methods

### Plant materials and isolation of KF

To obtain an ethanol extract, 2.5 kg of dried persimmon leaves was extracted with hot water at 65 °C for 3 h with 100% ethanol, followed by filtration and concentration under reduced pressure; 177.2 g of extract was obtained. Thereafter, the ethanol extract was suspended in distilled water and fractionated using *n*-hexane, chloroform, and ethyl acetate to obtain fractions of 23.7, 50.1, and 10.0 g, respectively. Next, silica gel column chromatography was performed on 10.0 g of the ethyl acetate fraction in CHCI_3_:MeOH (40:1 to 10:1) to obtain 5 subfractions (Fr.1 to Fr.5). Subsequently, 250.0 mg of compound **1** was obtained from fraction Fr.5 using a Sephadex LH-20 column (MeOH:H_2_O = 4:6; Sigma-Aldrich, St. Louis, MO, USA). Thereafter, nuclear magnetic resonance (NMR) analysis was performed on the separated compound to identify compound **1**, which was confirmed to be KF by comparison with previous studies [8]. The results of the NMR analysis were as follows: KF, yellow powder; electrospray ionization mass spectrometry: *m*/*z* 285.1 (M–H)– (C_15_H_10_O_6_), ^1^H-NMR (CD_3_OD, 500 MHz) *δ*: 8.08 (2H, d, *J* = 11.5, 2.8 Hz, H-2′, H-6′), 6.90 (2H, dd, *J* = 9.8, 2.7 Hz, H-3′, H-5′), 6.38 (1H, d, *J* = 2.0 Hz, H-8), 6.16 (1H, d, *J* = 2.0 Hz, H-6). ^13^C-NMR (CD_3_OD, 500 MHz) *δ*: 176.0 (C-4), 164.3 (C-7), 161.2 (C-9), 159.2 (C-4′), 156.9 (C-5), 146.7 (C-2), 135.8 (C-3), 129.3 (C-2′, 6′), 122.4 (C-1′), 115.0 (C-3′, 5′), 103.2 (C-10), 97.9 (C-6), 93.1 (C-8).

### Collection of overlapping targets between KF and AD

First, to identify KF and its active targets, the canonical Simplified Molecular Input Line Entry System (SMILES) code C1=CC(=CC=C1C2=C(C(=O)C3=C(C=C(C=C3O2)O)O)O)O in SwissTargetPrediction (http://www.swisstargetprediction.ch) was used, retrieving 100 targets. Next, 1,678 genes related to AD were retrieved from GeneCards (https://www.genecards.org). Using Venn analysis, 38 overlapping targets were identified by matching the AD target with the KF action target. Finally, in order to minimize false-positive predictions and ensure the reliability of downstream analyses, among the 38 overlapping targets, 5 targets corresponding to the upper probability of 1.0 were identified via SwissTargetPrediction. The characteristics of KF were obtained from the Traditional Chinese Medicine Systems Pharmacology Database and Analysis Platform (https://ngdc.cncb.ac.cn/databasecommons/database/id/4096). According to previous reports, the threshold criteria for good absorption and slow metabolism after oral administration are based on oral bioavailability and drug-likeness (DL), with values not less than 30% and 0.18, respectively [29].

### Protein association, protein–protein interaction network construction, Gene Ontology enrichment analysis, and Kyoto Encyclopedia of Genes and Genomes pathway determination

For the 5 selected targets, protein network analysis was performed using the Search Tool for the Retrieval of Interacting Genes/Proteins (STRING) (https://string-db.org), a web-based analysis tool, to confirm their association with heme oxygenase-1 (HO-1), one of the detoxifying enzymes regulated by the nuclear translocation of Nrf2. The target showing the best correlation was selected as a candidate target for the control of AD by KF. The overlapping targets between KF and AD were inputted into the STRING database (https://string-db.org/). The Cytoscape software (version 3.9.1, https://cytoscape.org/; Cytoscape Consortium, San Diego, CA, USA) was used to construct and visualize the protein–protein interaction (PPI) network. Disconnected edges were hidden by default in the network settings. ShinyGO was utilized for the analysis of the potential target list. Enrichment of pathways or biological processes was assessed, with criteria set for pathway sizes ranging from 2 to 2,000, and a false discovery rate threshold of less than 0.05. Furthermore, the target list was submitted to the DAVID database (https://ngdc.cncb.ac.cn/databasecommons/database/id/3061) to perform Kyoto Encyclopedia of Genes and Genomes (KEGG) pathway enrichment analysis, where pathways with a *P* value of less than 0.05 were considered significantly enriched, facilitating the identification of key biological pathways.

### Computational docking

The structure of KF was obtained using the PubChem database (https://pubchem.ncbi.nlm.nih.gov/compound/5280863). To prepare for the docking procedure, the 3-dimensional (3D) structure of KF was constructed using ChemDraw 20.1 and Chem3D 20.1 (PerkinElmer, Waltham, MA, USA) for 2-dimensional (2D) and 3D conformations, respectively. Computational docking analysis was performed using AutoDockTools 1.5.6 to simulate the binding mechanism and evaluate the binding energies of KF and potential targets according to previously described methods. The 3D structures of AHR, ATP binding cassette subfamily B member 1 (ABCB1), carbonic anhydrase 2 (CA2), arachidonate 5-lipoxygenase (ALOX5), and carbonic anhydrase 12 (CA12) were downloaded from the Protein Data Bank (PDB) with PDB IDs 7VNA, 7OTI, 1A42, 3O8Y, and 1JCZ, respectively. Interactions between KF and target proteins were analyzed according to binding affinity scores and root mean square deviation (RMSD). The 2D interaction graphics and 3D docking poses were displayed using BIOVIA Discovery Studio v21.1 (Dassault Systèmes, San Diego, CA, USA).

### Reagents and antibodies

3-(4,5-Dimethylthiazol-2-yl)-2,5-diphenyltetrazolium bromide (MTT), dinitrochlorobenzene (DNCB), 6-formylindolo[3,2-*b*]carbazole (FICZ), StemRegenin1 (SR1), and radioimmunoprecipitation assay buffer were purchased from Sigma-Aldrich (St. Louis, MO, USA). Recombinant human tumor necrosis factor α (TNF-α), interferon γ (IFN-γ), IL-4, and IL-13 were purchased from PeproTech (Rocky Hill, NJ, USA). Reagents for annexin V and caspase 3/7 staining were obtained from Essen Bioscience (Ann Arbor, MI, USA). The RT PreMix kit was purchased from Enzynomics (Daejeon, Korea). House dust mite (*Dermatophagoides farinae*) extract was obtained from Greer (Lenoir, NC, USA). Mouse IgE enzyme-linked immunosorbent assay kits were purchased from R&D Systems (Minneapolis, MN, USA). Protopic ointments (0.3%) were obtained from Johnson & Johnson (New Brunswick, NJ, USA). Antibodies against β-actin and extracellular signal-regulated kinase were purchased from Santa Cruz Biotechnology (Dallas, TX, USA). Antibodies against AhR, ARNT, and HO-1 were purchased from Cell Signaling Technology (Danvers, MA, USA). Antibodies against filaggrin and involucrin were purchased from Abcam (Cambridge, UK).

### Cell culture

HaCaT keratinocytes were kindly provided by Professor Eun-Kyung Kim (Department of Food Science and Nutrition, Dong-A University, Busan, Korea), and were originally obtained from the American Type Culture Collection (Manassas, VA, USA; cat. no. PCS-200-011). To ensure the integrity of the cell line and eliminate potential contamination, regular mycoplasma testing was performed throughout the study using the MycoAlert detection kit (TaKaRa, Tokyo, Japan). No signs of mycoplasma contamination were observed based on morphological monitoring using the Incucyte live-cell imaging system. Cells were cultured in Dulbecco’s modified Eagle medium (Welgene, Gyeongsan-si, Republic of Korea) supplemented with 10% fetal bovine serum, 100 units/ml penicillin G, 100 μg/ml streptomycin, and 2 mM l-glutamine. HaCaT cells were used between passages 3 and 8 and were maintained in a humidified incubator at 37 °C with 5% CO_2_ and 95% air.

### Cell viability

Cell viability was evaluated using MTT and live-cell assays and trypan blue staining. Briefly, HaCaT cells were incubated for 24 h at 37 °C with the indicated concentrations of KF (5 to 40 μM). After treatment with MTT (5 mg/ml) for 4 h, the cell culture plate was centrifuged, after which the supernatant was discarded, and 150 μl of dimethyl sulfoxide was added to each well to dissolve formazan crystals. Next, the absorbance was measured using a plate reader at a wavelength of 540 nm and the absorbance of each sample was compared with that of the control and expressed as a percentage (% of the control). In addition, the number of viable cells was measured using the Incucyte Live-Cell Analysis System (Sartorius, Göttingen, Germany). For the trypan blue assay, trypan blue solution (0.4%) was added to 1%. Cells loaded in 10 μl of the cell suspension were subjected to hemocytometry to calculate the percentages of viable (unstained) and dead (stained) cells and total cell number.

### Western blot analysis

Cells were lysed in lysis buffer (1% Triton X-100, 150 mM NaCl, 20 mM Tris [pH 7.5], 1 tablet protease inhibitor, and 1 tablet phosphatase inhibitor) on ice for 30 min and centrifuged at 14,000 rpm for 15 min. To separate the cytosolic and nuclear extracts, cell lysis was performed using NE-PER Nuclear and Cytoplasmic Extraction Reagents (Thermo Fisher Scientific, Waltham, MA, USA). The Pierce Bradford Protein Assay Kit (Thermo Fisher Scientific) was used to quantify the protein concentration according to the manufacturer’s instructions. Thereafter, following protein denaturation, approximately 40 μg of extract lysate was separated on 8% to 12% sodium dodecyl sulfate–polyacrylamide gel electrophoresis (SDS-PAGE) gels. After electrophoresis, the separated proteins were transferred to a polyvinylidene difluoride membrane, blocked in skim milk (1 h), rinsed, and incubated with the indicated primary antibodies in Tris-buffered saline containing 0.1% Tween 20 (TBS-T) overnight. Subsequently, the membranes were washed with TBS-T and incubated with the secondary antibodies at room temperature. Antibody-bound proteins were visualized using an electrochemiluminescence western blotting detection reagent (Thermo Fisher Scientific) and the ChemiDoc imaging system (Bio-Rad Laboratories, Hercules, CA, USA).

### Real-time quantitative PCR

Real-time polymerase chain reaction (PCR) was used to determine gene expression levels, and total RNA was extracted from cell lysates using TRIzol reagent (Thermo Fisher Scientific, Waltham, MA USA). Next, the messenger RNA (mRNA) concentration was analyzed using NanoDrop Spectrophotometer (Thermo Fisher Scientific) and TOPscript RT DryMIX (dT18 plus) (Enzynomics, Inc., Daejeon, South Korea). Reverse transcription of total RNA was performed using the RT PreMix kit (Enzynomics, Inc.). Gene expression was calculated using the 2^−ΔΔCT^ method, where ΔΔCT = (CTtarget − CTgapdh) at time *x* − (CTtarget − CTgapdh) at time 0. Here, time *x* represents the experimental time point, and time 0 represents the baseline (1×) indicating that genes in the untreated group were normalized to glyceraldehyde-3-phosphate dehydrogenase (GAPDH). The experiments were performed independently 3 times. The primers used in this study are in the Supplementary Materials (Table S1).

### Pull-down assay

To determine protein binding to KF, a KF–Sepharose 4B combination was generated as described previously [8]. Briefly, activated dried Sepharose 4B powder (GE Healthcare, Chicago, IL, USA) in 1 mM HCl was mixed with 2 mg of KF in a coupling buffer (0.1 M NaHCO_3_ [pH 8.3] and 0.5 M NaCl). After overnight mixing, the coupling buffer was removed and replaced with 0.1 M Tris–HCl buffer (pH 8.0). This complex was rotated overnight and washed once with 0.1 M acetate buffer (pH 4.0) containing 0.5 M NaCl, followed by a second wash with 0.5 M NaCl. Afterward, lysate from HaCaT cells stimulated with anti-CD3/CD28 antibodies for 24 h was incubated overnight with this complex or control Sepharose 4B beads in reaction buffer (50 mM Tris, 5 mM EDTA, 150 mM NaCl, 1 mM dithiothreitol, 0.01% NP-40, 2 mg/ml bovine serum albumin, 0.02 mM phenylmethylsulfonyl fluoride, and 1 μg of protease inhibitor). After rotation, the lysate–bead complex was washed with washing buffer (50 mM Tris, 5 mM EDTA, 150 mM NaCl, 1 mM dithiothreitol, 0.01% NP-40, and 0.02 mM phenylmethylsulfonyl fluoride) and eluted in SDS loading buffer. After gel loading, AhR was determined by western blotting using an anti-AhR antibody.

### Immunofluorescence assays

HaCaT cells were cultured and treated with the indicated concentrations of KF for 24 h, washed with phosphate-buffered saline (PBS), and fixed with 3.7% formaldehyde (Panreac, Barcelona, Spain). They were then permeabilized with 0.1% Triton X-100 in PBS, and AhR (Thermo Fisher Scientific, Waltham, MA, USA) antibody was diluted (1:100). They were then washed with PBS and incubated with specific anti-IgG secondary antibodies conjugated to Alexa Fluor 488 (Invitrogen, Waltham, MA, USA). After incubation, images were captured using the Incucyte Live-Cell Analysis System (Sartorius, Göttingen, Germany).

### Cellular thermal shift assay

Cells were cultured and treated with KF (40 μM) or dimethyl sulfoxide as a control for 1 h. Following treatment, cells were washed twice with PBS and lysed using radioimmunoprecipitation assay buffer. The lysates were centrifuged at 15,000 rpm for 15 min at 4 °C to collect the supernatant. Each sample was then divided into 5 equal aliquots and subjected to a temperature gradient ranging from 37 to 69 °C, in 8 °C increments. After heating at the designated temperatures for 3 min, samples were immediately cooled on ice for 3 min and centrifuged to remove precipitated proteins. The resulting supernatants were transferred to fresh tubes, mixed with 5× SDS-PAGE loading buffer, and boiled for 10 min. Protein stability was subsequently evaluated by immunoblotting with an AhR antibody.

### Prediction of DL and absorption, distribution, metabolism, excretion, and toxicity

Drug likeliness properties and absorption, distribution, metabolism, excretion, and toxicity (ADMET) predictions were performed using Accelrys DS 4.0 (Accelrys Software Inc.) and were performed on the 3D conformation (KF [CID: 5280863]). The binding of all compounds was evaluated with the Lipinski fusion filter implemented in DS 4.0, and pharmacokinetic properties were predicted using information on the configuration of the ligand as a molecule and the ADMET treatment, explaining the physical performance properties. The pharmacokinetic ring of KF was performed using TOPKAT (Computer Aided Technology to Monitor) used in DS 4.0, and TOPKAT was analyzed using the quantitative structure–toxicity relationship model to evaluate various functional measures of functions that depend only on the accessory structure.

### Preparation of the AD-like reconstructed human skin model

The reconstructed human skin (RHS) model is a model created by reconstructing human epidermis by inducing differentiation of keratinocytes that form the epidermis of human skin, and the RHS model (Neoderm-ED) used in the study was purchased from TEGO Science Inc. (Seoul, Korea). In order to induce AD in the RHS model, the cells were treated with or without the indicated concentration of KF (20 and 40 μM) for 6 d while being treated with an inflammatory cocktail (AD cocktail consisting of IL-4, 30 ng/ml; IL-13, 30 ng/ml; and TNF-α, 3.5 ng/ml). The composition and concentration of the inflammatory cocktail used in this study were based on previously reported studies. IL-4 and IL-13 are known to induce Th2-mediated responses in keratinocytes, while TNF-α amplifies inflammatory signals, mimicking AD [30,31]. During the 6-d culture, the culture medium was replaced every day and treated in the same way. The entire experimental method was performed according to a previously described method [32].

### Mice

Six-to-eight-week-old female BALB/c mice were obtained from Samtako (Osan, Republic of Korea) and housed under specific-pathogen-free conditions. All experiments were approved by the Animal Care and Use Committee of the College of Pharmacy, Chungnam National University (approval number: 202212-CNU-258).

### Induction of AD

Four treatments were applied: naive mice that were not treated with either DNCB/mite extract or KF (control, *n* = 5), control mice treated with DNCB/mite extract alone (AD group, *n* = 5), experimental mice simultaneously treated with DNCB/mite extract and KF (AD+KF group, *n* = 5), and positive control mice simultaneously treated with DNCB/mite extract and Protopic (AD+Protopic group, *n* = 5). AD in BALB/c mice was induced by repeated application of the mite extract and DNCB on the ears of mice, as described previously [33]. Briefly, the surfaces of both ears were stripped 5 times with surgical tape (Seo-il Chemistry, Hwasung, Korea). After stripping, each ear was applied with 20 μl of DNCB (1%). Four days later, ears were painted with 20 μl of mite extract (10 mg/ml). The mite extract/DNCB treatment was repeated weekly for 4 weeks. Treatment was started 1 d after the second DNCB application. This was repeated daily for 4 subsequent days. After a 2-d break, the 5-d-on and 2-d-off KF oral administration protocol was repeated for 4 weeks. Ear thickness was evaluated 24 h after applying DNCB or mite extract using a dial thickness gauge (Kori Seiki Mfg. Co., Ltd., Tokyo, Japan). At the end of the experiment after 28 d, the experimental animals were sacrificed through 70% CO_2_ inhalation.

### Histological analysis

After sacrifice on the 28th day after induction of AD, the ears were removed for histopathological analysis. To eliminate biased results, one animal from each of the 5 experimental groups was randomly assigned to the same site and stained. The ears were fixed with 10% paraformaldehyde and embedded in paraffin. The paraffin-embedded ears were sectioned at a 5-μm thickness, deparaffinized, and stained with hematoxylin and eosin (H&E). Dermal and epidermal thicknesses were measured on H&E-stained slides. To identify infiltrating mast cells, sections were stained with 0.01% toluidine blue and mast cells were stained at 5 randomly selected sites. The sites were then aggregated.

### Statistics

All data are expressed as mean ± standard deviation. For *in vitro* experiments, statistical analyses were conducted using data from at least 3 independent biological replicates performed on separate days. For *in vivo* experiments, data were obtained from 5 mice per group. One-way analysis of variance followed by Tukey’s post hoc test was used for multiple-group comparisons, and unpaired 2-tailed Student *t* tests were used for pairwise comparisons. A *P* value less than 0.05 was considered statistically significant. Sample sizes (*n*) and exact *P* values are indicated in the figure legends.

## Results

### Identification of potential overlapping targets on KF and AD through PPI network construction

**Fig. 1.**
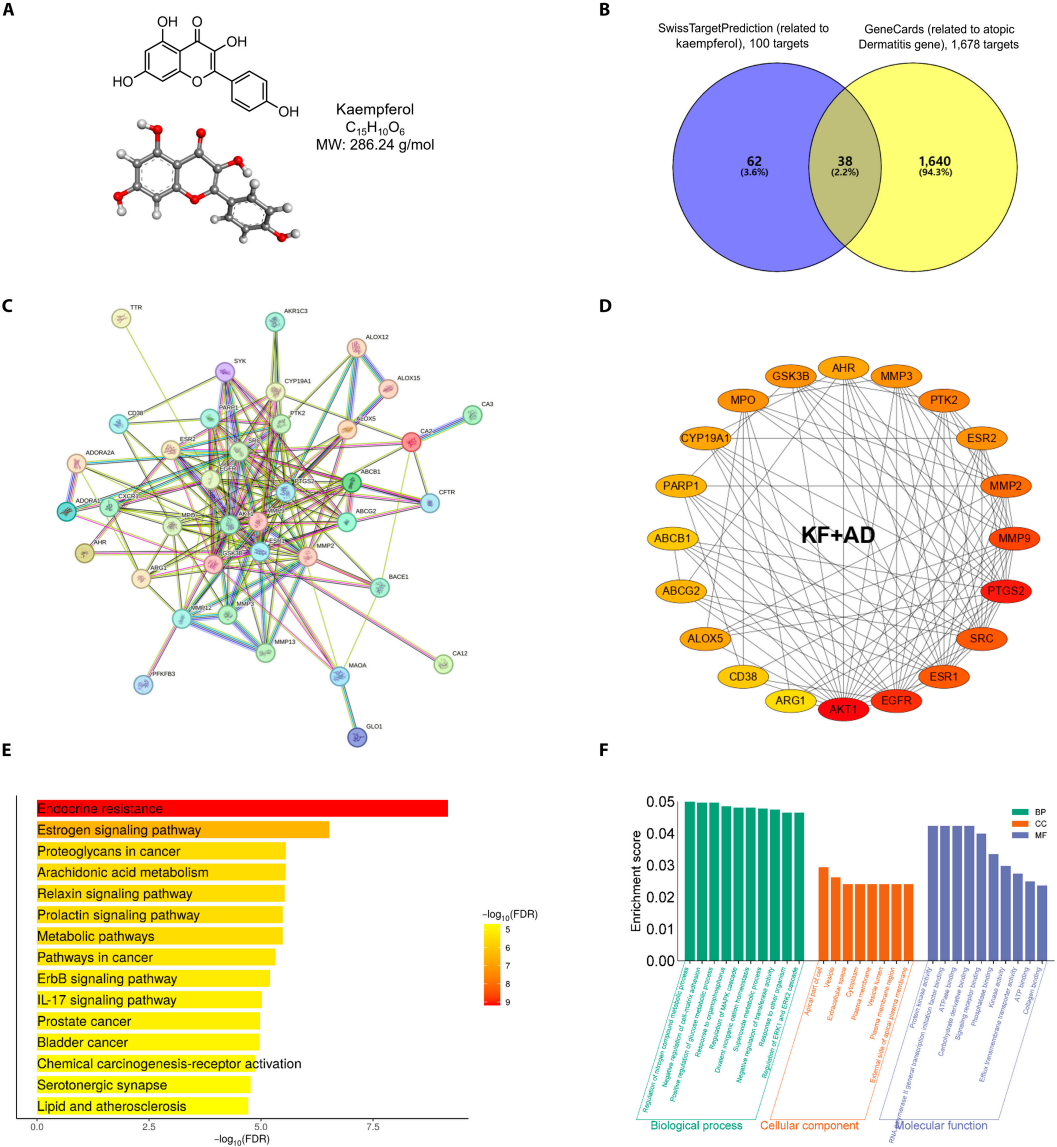
Kaempferol (KF)-associated atopic dermatitis (AD) target protein association network pharmacology. (A) Chemical structure of KF in 2 dimensions and 3 dimensions. (B) Exploration of overlap genes related to AD and KF through Venn analysis. (C) String analysis of overlap genes. (D) Top 20 targets ranked by the degree method. Interaction between KF and associated residues of aryl hydrocarbon receptor (AhR) protein constructed by Discovery Studio (v21.1, Dassault Systèmes, San Diego, CA, USA) in 3 dimensions and 2 dimensions. (E) Kyoto Encyclopedia of Genes and Genomes (KEGG) analysis. (F) Gene Ontology (GO) enrichment. MW, molecular weight; IL-17, interleukin-17; FDR, false discovery rate.

To establish the relationship between KF (canonical SMILES: C1=CC(=CC=C1C2=C(C(=O)C3=C(C=C(C=C3O2)O)O)O) (Fig. 1A) and AD, 100 KFs were identified using the SwissTargetPrediction database (http://www.swisstargetprediction.ch). Based on the GeneCards database (https://www.genecards.org), 1,678 potential targets related to AD were identified. In total, 38 overlapping targets between KF- and AD-associated genes were identified as key candidates for anti-AD effects using Venny 2.1.0 tools (https://bioinfogp.cnb.csic.es/tools/venny) (Fig. 1B and Table S2). The compound–gene target network was constructed using the STRING database (https://string-db.org/) to provide the relevant protein–protein associations before sending them to Cytoscape (v.3.8.2, Institute for Systems Biology, USA) to examine the relationship between KF and gene targets in AD (Fig. 1C). According to the top 20 targets based on the degree-method-generated PPI network along with the close-to-probability-1 targets retrieved from the SwissTargetPrediction database, the final targets to investigate anti-AD were identified, including ABCB1, CA2, ALOX5, CA12, and AhR (Fig. 1D and Tables S3 and S4).

KEGG pathway analysis revealed 15 significantly enriched pathways. Among those, several pathways are implicated in the pathophysiology of AD. Specifically, the IL-17 signaling pathway drives inflammation and skin barrier dysfunction, while arachidonic acid metabolism contributes to the inflammatory cascade through prostaglandins and leukotrienes. Additionally, estrogen signaling modulates immune responses and skin barrier integrity, particularly in women, influencing AD severity. These pathways are central to the chronic inflammation and barrier disruption observed in AD (Fig. 1E). Following GO enrichment analysis, 3 categories (biological process, cellular component, and molecular function) were detected. The 10 most significantly enriched terms in each category are presented in Fig. 1F.

### Molecular docking result and validation

**Fig. 2.**
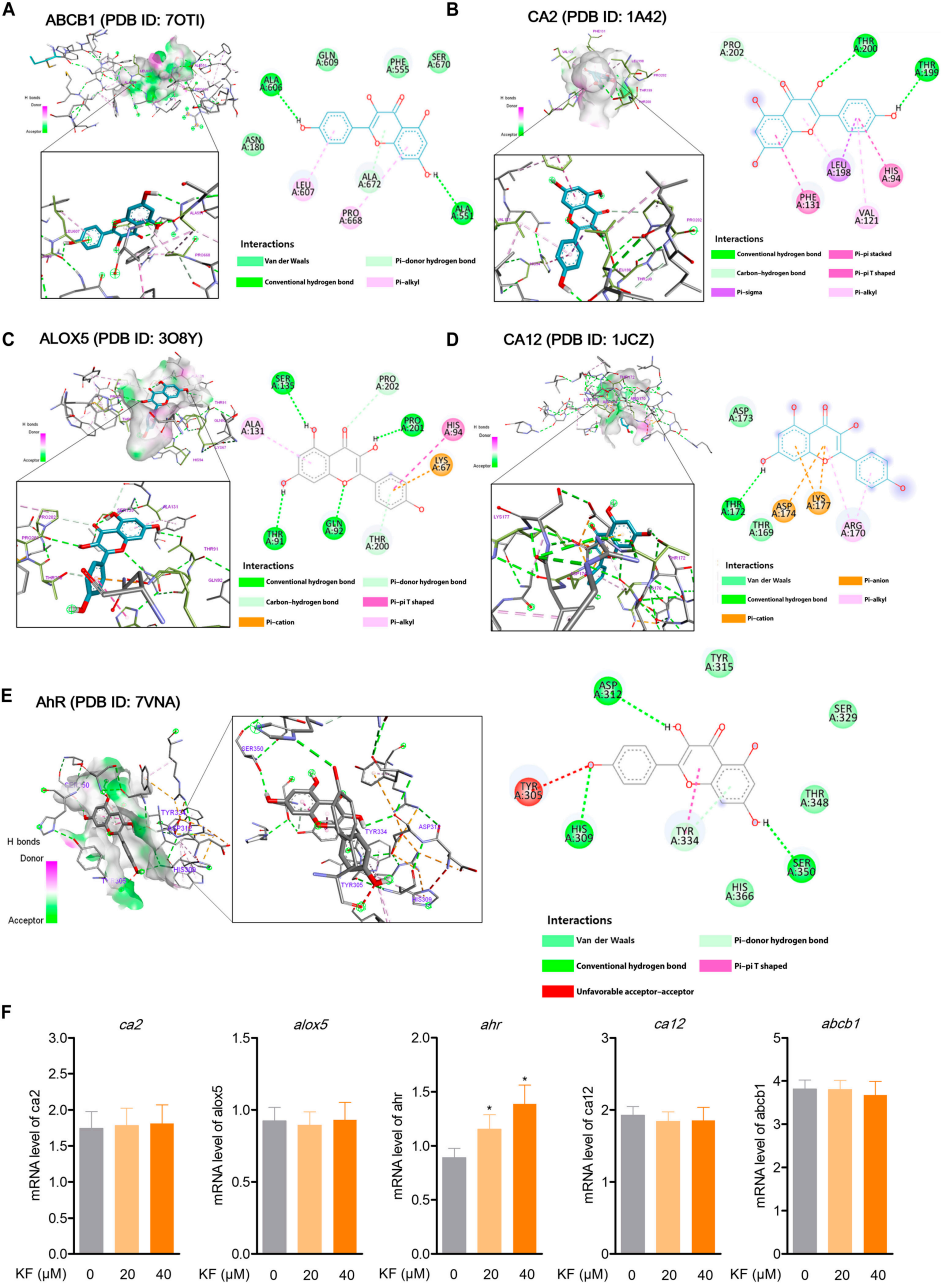
The 3-dimensional (3D) docking pose and 2-dimensional (2D) interaction diagrams of KF with potential targets. (A to E) Interactions between KF and associated residues in the interface of arachidonate 5-lipoxygenase (ALOX5; Protein Data Bank [PDB] ID: 3O8Y), carbonic anhydrase 2 (CA2; PDB ID: 1A42), carbonic anhydrase 12 (CA12; PDB ID: 1JCZ), ATP binding cassette subfamily B member 1 (ABCB1; PDB ID: 7OTI), and AhR proteins (PDB ID: 7VNA) were generated using Discovery Studio (v21.1, Dassault Systèmes, San Diego, CA, USA). (E) KF interactions with AhR interface residues (PDB ID: 7VNA). (F) HaCaT cells were seeded at 5 × 10^3^ cells/ml, cultured for 24 h, and then treated with the indicated concentrations of KF (20 and 40 μM), and then the messenger RNA (mRNA) levels of the indicated genes were evaluated using real-time quantitative polymerase chain reaction (qPCR). The results represent the mean ± SD of 3 independent experiments (*n* = 3). *P < 0.05 versus the control group (KF 0 μM).

For the 5 selected targets, ABCB1, CA2, ALOX5, CA12, and AhR, molecular docking simulation was conducted using AutoDockTools 1.5.6 to evaluate the binding efficacy and key amino acid interactions with KF. The virtual structures of ABCB1 (PDB ID: 7OTI), CA2 (PDB ID: 1A42), ALOX5 (PDB ID: 3O8Y), CA12 (PDB ID: 1JCZ), and AHR (PDB ID: 7VNA) were selected as receptor structures for virtual screening according to previously reported fundamental studies (Fig. 2A to E). Among the 5 targets, AhR (7VNA) showed the highest binding energy with KF, and the data showed that it had the highest binding energy and RMSD values for KF of −7.6 kcal/mol and 1.83 Å, respectively. In addition, ALOX5 was next, with an RMSD of −7.4 kcal/mol, and those of CA2 and CA12 were −6.3 kcal/mol and that of ABCB1 was 4.4 kcal/mol, each showing the same binding affinity to KF (Table S5). Therefore, AhR was selected as the AD regulatory target showing the highest binding affinity to KF. In addition, in order to compare the specific affinity for KF for AhR, which was discovered as the AD target of KF, we measured the binding affinity to tapinarof, a representative natural-product-derived treatment currently used for AD or skin inflammation, and the measurement results were as follows: A lower result than that of KF was confirmed with a binding force of 7.1 kcal/mol (Table S6). These results suggested that AhR would show the best target for KF in the regulation of AD. To validate the *in silico* computational docking data, the cellular thermal shift assay was carried out to directly measure the degree of KF binding to AhR; as illustrated in Fig. 2E (Fig. S1), KF markedly increased the thermal stability of the AhR protein, suggesting a direct binding interaction between KF and AhR. When we evaluated the effect of KF treatment on these top 5 targets in actual HacaT cells, we found that KF increased the AhR gene level (Fig. 2F).

### KF is not cytotoxic in HaCaT cells and activates the AhR signaling pathway

**Fig. 3.**
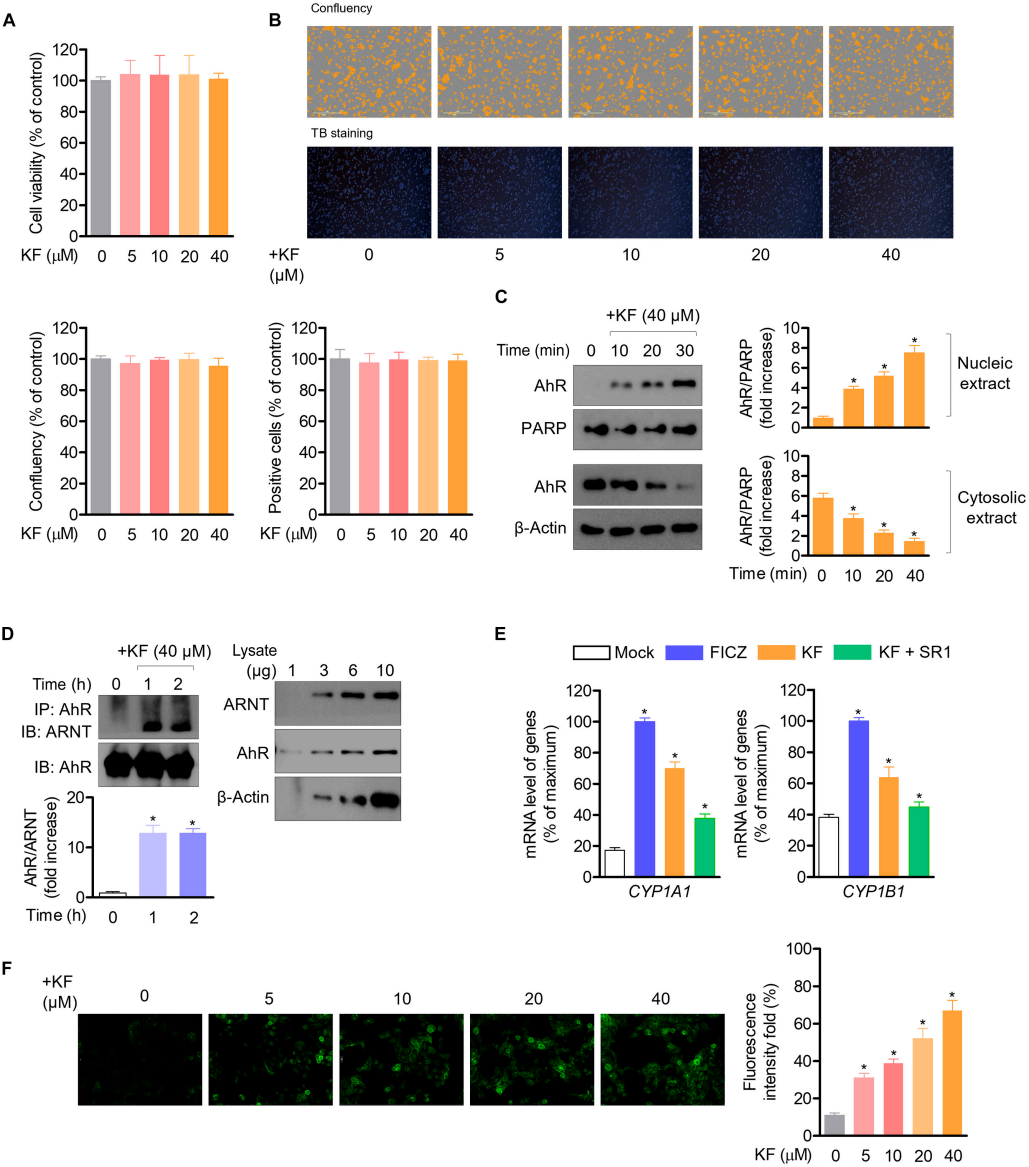
KF is noncytotoxic in HaCaT cells and stimulates the AhR signaling pathway. HaCaT cells were seeded at 5 × 10^3^ cell/ml into 96-well plates, cultured for 24 h, and then treated with the indicated concentration of KF (5 to 40 μM). Cell toxicity and confluency were assessed using the 3-(4,5-dimethylthiazol-2-yl)-2,5-diphenyltetrazolium bromide (MTT) assay and IncuCyte live-cell assay system. (A and B) KF did not show cytotoxicity in HaCaT cells. (C) AhR translocation was detected using western blotting in HaCaT cells treated with KF (40 μM) for 0 to 30 min. (D) The aryl hydrocarbon receptor nuclear translocator (ARNT) bound to AhR in HaCaT cells treated with KF (40 μM) for 0 to 2 h was detected using a co-immunoprecipitation assay. HaCaT cells were treated with 6-formylindolo[3,2-*b*]carbazole (FICZ; 100 nM), StemRegenin1 (SR1; 1 μM), and/or KF (40 μM) for 24 h. (E) The mRNA levels of indicated genes were evaluated using qPCR. (F) AhR expression was analyzed by immunofluorescence staining. Results are expressed as mean ± standard error of the mean (SEM) (*n* = 3). *P < 0.05 versus the control group (KF 0 μM). TB, trypan blue; PARP, poly ADP-ribose polymerase; IP, immunoprecipitation; IB, immunoblotting.

We determined whether KF was cytotoxic in HaCaT cells. MTT and apoptosis assays revealed that KF did not result in any cellular toxicity or apoptosis up to a concentration of 40 μM (Fig. 3A). In addition, in the analysis using the live-cell system, KF did not affect cell counting, no morphological changes in cells occurred, and trypan blue staining confirmed that KF treatment had no effect on cell survival (Fig. 3B). Complementary DNA microarray analysis was used to predict KF-binding partners, revealing that KF bound to various transcription factors, including AhR, in keratinocytes. To determine whether KF treatment induces the translocation of AhR into the nucleus in HaCaT cells, AhR expression was measured in the nuclear and cytosolic fractions after KF treatment. Cytosolic AhR gradually translocated into the nucleus in a time-dependent manner (Fig. 3C). An immunoprecipitation assay was performed to investigate whether KF enhances the AhR–ARNT interaction in HaCaT cells. The interaction between AhR and ARNT was significantly augmented in cells treated with 40 μM KF (Fig. 3D). To explore whether enhanced heterodimer formation by KF promoted AhR activity, the mRNA levels of *CYP1A1* and *CYP1B1*, genetic markers typically induced by the AhR pathway, were determined by quantitative PCR. KF increased the expression levels of *CYP1A1* and *CYP1B1* in HaCaT cells (Fig. 3E), similarly as did FICZ, an AhR ligand. Cotreatment with SR1, an AhR inhibitor [34], abolished the effect of KF on the induction of AhR signaling. In addition, it was confirmed through immunocytochemical staining that KF induces the expression of AhR in HaCaT cells (Fig. 3F).

### KF enhances antioxidant effects via the Nrf2 pathway and promotes terminal epidermal differentiation through AhR activation in HaCaT cells

**Fig. 4.**
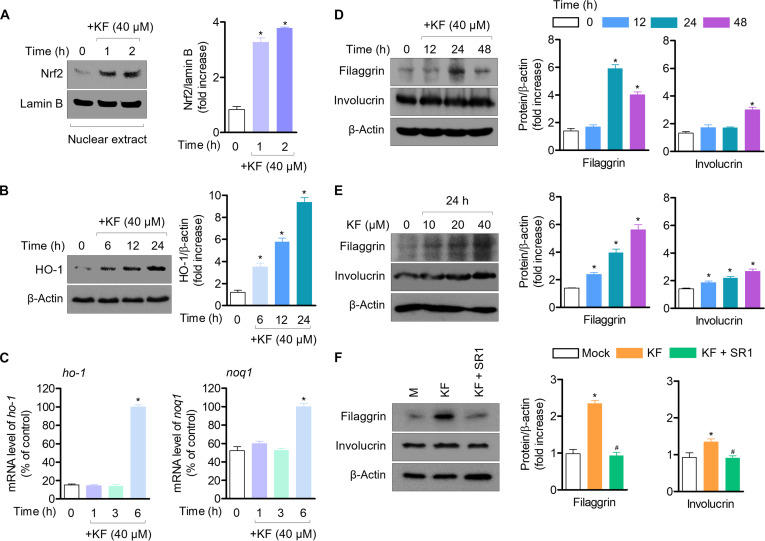
KF exerts antioxidant effects through the nuclear factor erythroid 2-related factor 2 (Nrf2) pathway in HaCaT cells and enhances epidermal terminal differentiation by inducing the AhR pathway in HaCaT cells. (A) HaCaT cells were seeded at 5 × 10^5^ cell/ml into 6-well plates; then, after, HaCaT cells were pretreated with KF (40 μM) for 2 h and Nrf2 translocation was detected using western blotting. Nrf2 (nucleus) and lamin B were detected as loading control. (B) Western blot of heme oxygenase-1 (HO-1) expression after treatment of HaCaT cells with KF (40 μM) for 6, 12, and 24 h. (C) The mRNA levels of the indicated genes were evaluated by qPCR. (D and E) To evaluate the expression of epidermal differentiation proteins (filaggrin and involucrin), KF (40 μM) was treated for 12, 24, and 48 h and confirmed through western blotting analysis. (F) Epidermal differentiation protein expression was evaluated by pretreatment with SR1 (1 μM) and KF (40 μM) for 1 h. The proteins were detected using western blotting. Results are expressed as mean ± SEM (*n* = 3). *P < 0.05 versus the control group (KF 0 μM); ^#^*P* < 0.05 versus the KF-only-treated group.

As AhR activation promotes the Nrf2 pathway, which is significantly involved in cytoprotective effects [35], we evaluated HO-1 expression and Nrf2 translocation. First, treatment with KF induced the nuclear translocation of Nrf2, which resulted in a time-dependent increase in HO-1 protein expression (Fig. 4A and B). Accordingly, treatment with KF also up-regulated the *ho-1* gene level and *noq1* gene expression, an indicator of Nrf2 activation (Fig. 4C). Also, to understand whether elevated AhR activity induced by KF increases the expression of genes involved in epidermal terminal differentiation, the expression of filaggrin and involucrin was detected using western blotting. KF treatment up-regulated filaggrin and involucrin expression in HaCaT cells (Fig. 4D and E), but the up-regulation of epidermal terminal differentiation proteins by KF was inhibited by treatment with the AhR antagonist SR1 (Fig. 4F). These results indicate that enhanced AhR activity by KF treatment promotes its interaction with ARNT and, subsequently, induces the transcription of genes associated with epidermal terminal differentiation in HaCaT cells.

### KF regulates HO-1 and epidermal terminal differentiation proteins through the AhR pathway in HaCaT cells

**Fig. 5.**
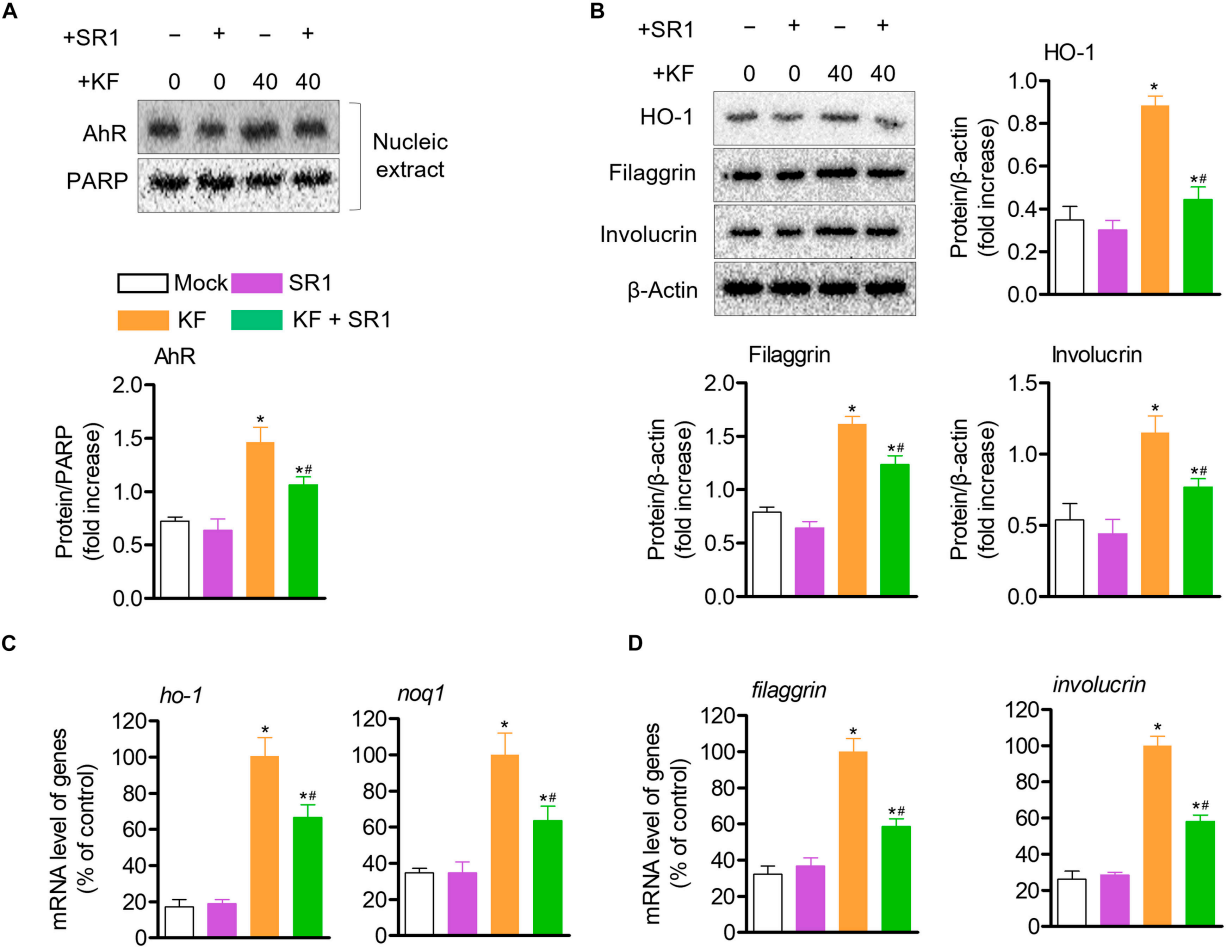
KF regulates HO-1 and epidermal terminal differentiation proteins through the AhR pathway in HaCaT cells. (A) HaCaT cells were seeded at 5 × 10^5^ cell/ml into 6-well plates; then, after, HaCaT cells pretreated with SR1 (1 μM) for 1 h and treated with KF (40 μM) for 24 h. AhR and PARP (nucleus) were detected using western blotting. (B) HO-1, filaggrin, and involucrin were also detected through western blotting by pretreating with SR1 (1 μM) for 1 h and then treating with KF (40 μM) for 24 h. (C and D) Similarly, after pretreatment with SR1 for 1 h and treatment with KF (40 μM) for 6 h, the mRNA levels of *ho-1* and *noq1* were analyzed using qPCR. Results are expressed as mean ± SEM (*n* = 3). *P < 0.05 versus the control group (KF 0 μM); ^#^*P* < 0.05 versus the KF-only-treated group.

As shown in Fig. 4, KF induced the expression of HO-1 through the translocation of Nrf2 into the nucleus; it was confirmed that filaggrin and involucrin, transcription proteins related to epidermal terminal differentiation increased by KF treatment, were suppressed by treatment with SR1, an inhibitor of AhR. These results suggested that the effect of KF on regulating epidermal terminal differentiation proteins may be regulated by AhR. Therefore, to evaluate the impact of AhR regulation on HO-1 and epidermal terminal differentiation proteins, we treated the cells with SR1 and evaluated the effects on the expression levels of these proteins and genes. The results of the study showed that KF treatment in keratinocytes similarly induced the protein expression of AhR and HO-1, and the expression of epidermal terminal differentiation proteins was also increased. However, it was confirmed that treatment with SR1 reversed the inducing effect of KF on HO-1 expression (Fig. 5A). In addition, treatment with SR1 was shown to reverse the effect of KF on filaggrin and involucrin protein expression shown previously (Fig. 5B). Likewise, it was confirmed that the antioxidant effect of KF in regulating the gene expression levels of *ho-1* and *noq1* was reversed by SR1 treatment, and the results of the epidermal terminal differentiation genes filaggrin and involucrin were also reversed (Fig. 5C and D). Taken together, these results suggest that the effects of KF on inducing epidermal filaggrin, involucrin, and HO-1 expression may be due to regulation by AhR. Furthermore, to verify that this reversal effect was attributable to the off-target regulation of SR1, we examined the protein expression of these factors following AhR silencing. Consistently, silencing of AhR abolished the KF-mediated regulatory effects on HO-1, filaggrin, and involucrin expression (Fig. S2).

### KF regulates the expression of inflammatory cytokines and epidermal terminal differentiation proteins through AhR regulation

**Fig. 6.**
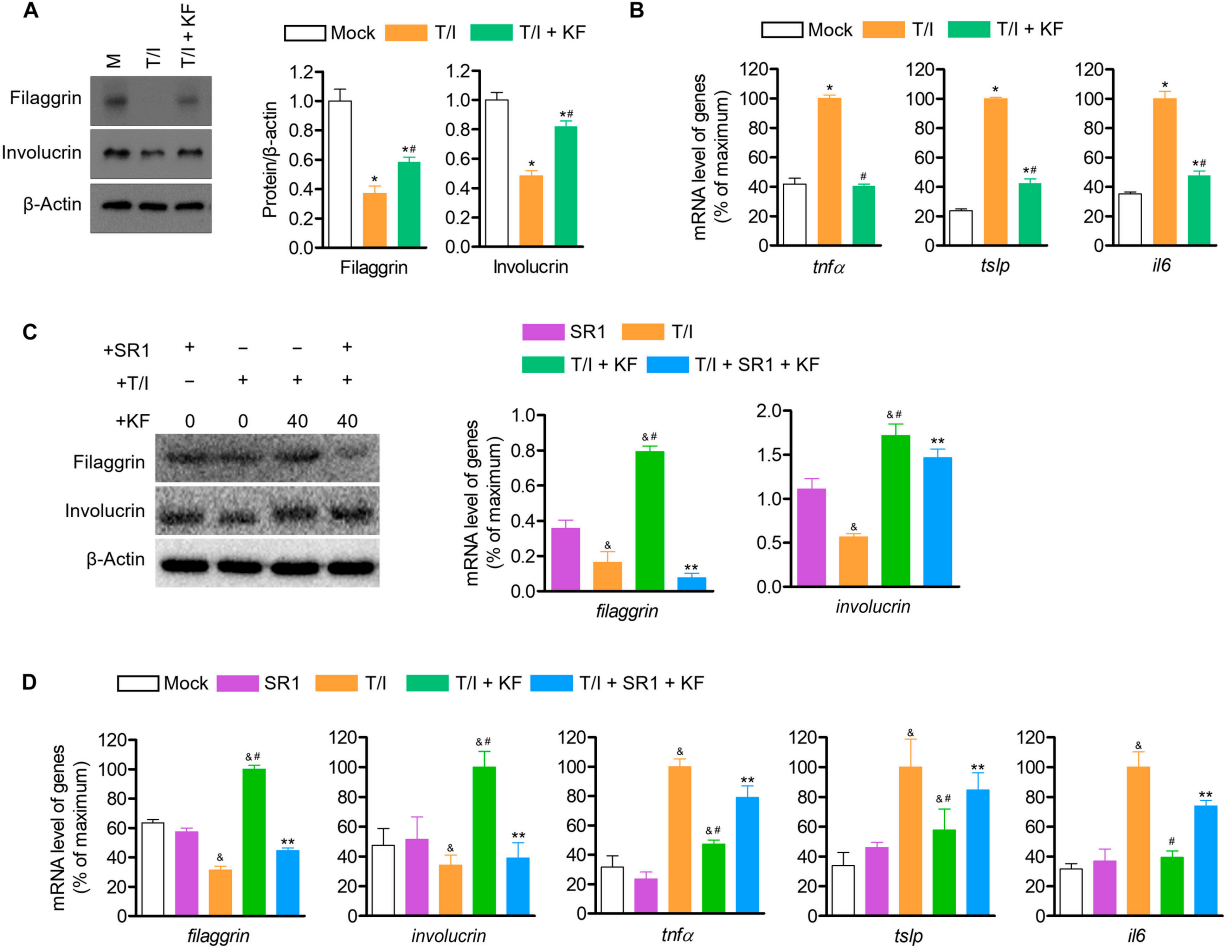
KF down-regulates the expression of pro-inflammatory cytokines and epidermal terminal differentiation of HaCaT cells activated by tumor necrosis factor α (TNF-α)/interferon γ (IFN-γ) through AhR regulation. (A) HaCaT cells were seeded into 6-well plates at a concentration of 5 × 10^5^ cells/ml. Afterward, cells were pretreated with KF (40 μM) for 1 h and stimulated with TNF-α (10 ng/ml)/IFN-γ (10 ng/ml) for 24 h. (B) In addition, SR1 (1 μM) was pretreated for 1 h and then treated with KF (40 μM) for 24 h to detect protein expression through western blot analysis. (C) HaCaT cells pretreated with KF (40 μM) for 1 h were stimulated with TNF-α (10 ng/ml)/IFN-γ (10 ng/ml) for 6 h for the detection of the mRNA levels of genes using qPCR. (D) In addition, after pretreatment with SR1 (1 μM), the cells were treated with KF (40 μM) for 1 h and then stimulated with TNF-α/IFN-γ for 6 h, and the mRNA levels of the genes were detected using qPCR. Results are expressed as mean ± SEM (*n* = 3). *P < 0.05 versus the control group (KF 0 μM); ^#^*P* < 0.05 versus the only T/I treat group; ^&^*P* < 0.05 versus the only SR1 treat group; **P < 0.05 versus the T/I + KF-only-treated group. T/I, TNF-α/IFN-γ.

A recent report has demonstrated the anti-inflammatory role of AhR-mediated pathways in human keratinocytes. Therefore, we evaluated the inflammatory control effect of KF and its effect on epidermal terminal differentiation proteins in keratinocytes stimulated with TNF-α/IFN-γ. First, the expression of the epidermal terminal differentiation proteins filaggrin and involucrin, which were lost in activated HaCaT cells stimulated with TNF-α/IFN-γ, was restored or protected by treatment with KF (quantifying recovery efficiency: filaggrin, 33%; involucrin 65%) (Fig. 6A). In addition, in the inflammation of keratinocytes, treatment with KF down-regulated the gene expression levels of *tnfα*, *tslp*, and *il6*, which were increased by TNF-α/IFN-γ (quantifying recovery efficiency: *tnfα*, 102%; *tslp*, 75%; *il6*, 80%) (Fig. 6B). Likewise, it was confirmed that these anti-inflammatory effects and the regulatory effects of epidermal terminal differentiation were reversed by SR1 treatment (Fig. 6C and D). Furthermore, after silencing AhR, we measured these inflammatory cytokines, confirming that the anti-inflammatory effect of KF was reversed (Fig. S3). These results ultimately suggest that the anti-inflammatory effect of KF is also exerted by AhR regulation and that AhR regulation by KF is also involved in regulating the expression levels of epidermal terminal differentiation proteins and pro-inflammatory cytokines.

### DL prediction and ADMET identification of KF

**Fig. 7.**
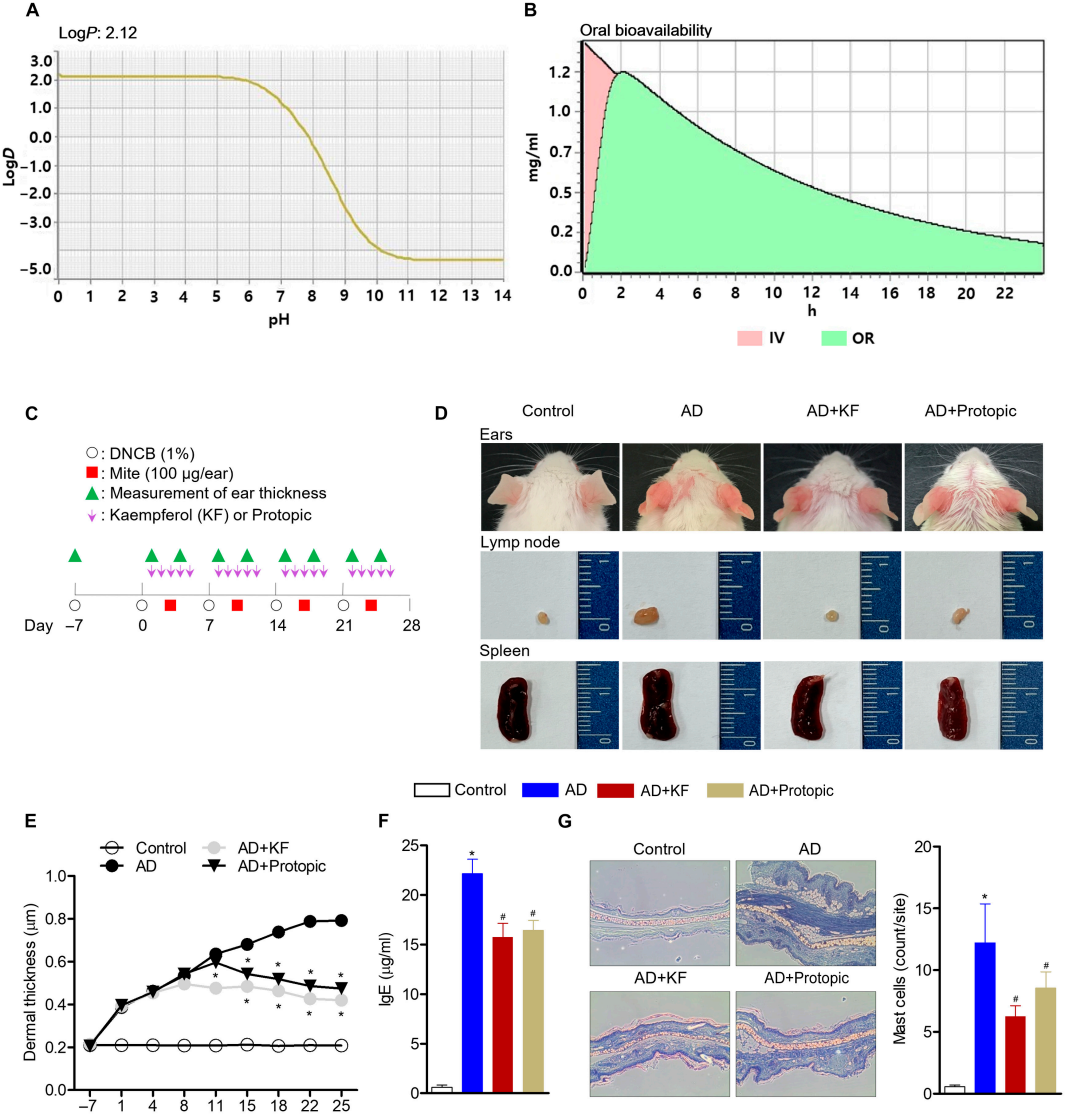
KF’s absorption, distribution, metabolism, excretion, and toxicity (ADMET) identification and its effect on relieving the symptoms of AD. (A) Prediction of the drug properties of KF according to Lipinski’s 5 laws. (B) Area under the curve (AUC) and bioavailability of KF. The oral administration of KF attenuates the symptoms of AD in mice. (C) Schematic diagram of animal experiments. (D) Representative images of each experimental group. Ear thicknesses were evaluated for 4 weeks. (E) Control, no treatment; AD, dinitrochlorobenzene (DNCB) and mite extract; AD+KF, AD mice orally administrated with 50 mg/kg of KF; AD+Protopic, AD mice orally administered with 10 μl of Protopic. (F) At 28 d post-induction of AD, serum immunoglobulin E (IgE) levels were measured using enzyme-linked immunosorbent assay (ELISA). (G) Representative pictures of toluidine blue-stained sections and the counts of infiltrating mast cells into ear tissues from each group. Results are expressed as the mean ± SEM of 5 mice (*n* = 5). *P < 0.05 versus the control group; ^#^*P* < 0.05 versus the only-AD-induced group. IV, intravenous injection; OR, oral administration.

In addition to the AD treatment results of KF shown above, we explored the DL of KF treatment efficacy when applied to the actual human body and predicted its usability as an actual drug. The prediction of KF drug properties was filtered by Lipinski’s rule of 5. The prediction results showed that KF had 4 or fewer hydrogen bond donors (expressed as the sum of all OH and NH groups), 6 or fewer hydrogen bond acceptors (expressed as the sum of all N’s and O’s), a molecular weight of less than 286.24 Da, and an octanol–water partition coefficient log*P* of less than 2.12 (Fig. 7A and Table S7). This is an excellent result that satisfies the criteria of Lipinski’s rule of 5: hydrogen bond donors (expressed as the sum of all OH and NH groups) should not exceed 5, hydrogen bond acceptors should not exceed 10 (expressed as the sum of all N’s and O’s), a molecular weight of less than 500 Da, and an octanol–water partition coefficient log*P* of less than 5. In addition, in order to confirm the possibility of clinical development application of KF, we confirmed drug-like properties and also predicted ADMET. The predicted results showed that KF has excellent human intestinal absorption, is chemically stable in acidic conditions, has very high intestinal absorption when administered orally, and shows drug characteristics such as lipid membrane permeability and good intercellular absorption. This means that it can exhibit maximum drug effect when administered orally. In addition, when administered orally at 50 mg, there is almost no first-pass effect in the liver and intestines, and the intestinal absorption is high, so the oral bioavailability is very high at 98.9% (Tmax: 125 min; Cp Max: 1.2 μg/ml). Among them, 93.54% of KF is predicted to bind mainly to human serum albumin (Fig. 7B), which means that it is distributed throughout the body. Previous studies have reported that several flavonoid compounds, including KF analogs, such as quercetin and genistein, exhibit extensive tissue absorption in the liver and intestines, suggesting that detection at plasma concentrations may be at very low doses [36]. Furthermore, KF is rapidly metabolized in the gastrointestinal tract and liver through extensive phase II metabolism (primarily glucuronidation, UDP-glucuronosyltransferase, sulfotransferase, and catechol-*O*-methyltransferase), approximately 81% of KF is metabolized via glucuronidation, and some glycoside forms are known to be detected in plasma after absorption [37]. In toxicity prediction, KF is a category 4 drug and is relatively safe with a similar level to aspirin (50 to 300 mg/kg) and acetaminophen (300 to 2,000 mg/kg) and carcinogenicity, DNA damage, and chromosomal aberration analysis. In this case, it was not shown to be positive. In addition, it was shown to be very unlikely to act as a P-glycoprotein substrate or inhibitor of efflux transporters that limit drug absorption in the intestines and central nervous system (Table S8). Therefore, in the subsequent *in vivo* study, KF was administered orally and the experiment was conducted.

### KF attenuates the symptoms of AD in mice

To explore whether the oral absorption of KF exhibits inhibitory effects on skin disease progression based on the results from HaCaT cells, we orally administered KF to an AD mouse model (Fig. 7C). AD symptoms improved in the AD group administered with KF compared with those in the AD group, and KF restored the size of enlarged lymph nodes and spleen, which are typical symptoms of AD (Fig. 7D). Furthermore, the AD group administered with KF showed reduced ear thickness, which is a marker of AD alleviation, compared with the AD group (Fig. 7E). IgE production was also measured to determine whether oral administration systemically influenced AD pathogenesis. The oral administration of KF suppressed serum IgE levels 28 d post-induction (quantifying recovery efficiency: IgE, 29%) (Fig. 7F). As mast cell infiltration into tissues occurs during AD development, sectioned ear tissues were stained with toluidine blue to identify penetrating mast cells. Ear sections from AD mice revealed increased numbers of mast cells in tissues compared with those in control mice; however, a significantly reduced number of mast cells was detected in AD mice following the oral administration of KF (quantifying recovery efficiency: mast cells, 50%) (Fig. 7G). Protopic application also decreased the number of mast cells in mice with AD, which correlated with ear thickness. These *in vivo* data suggest that the oral administration of KF effectively attenuates the progression of AD, reduces dermal and epidermal thickness, and enhances epidermal terminal differentiation.

### KF promotes terminal epidermal differentiation in ear tissue and exerts anti-inflammatory effects via AhR and HO-1 induction

**Fig. 8.**
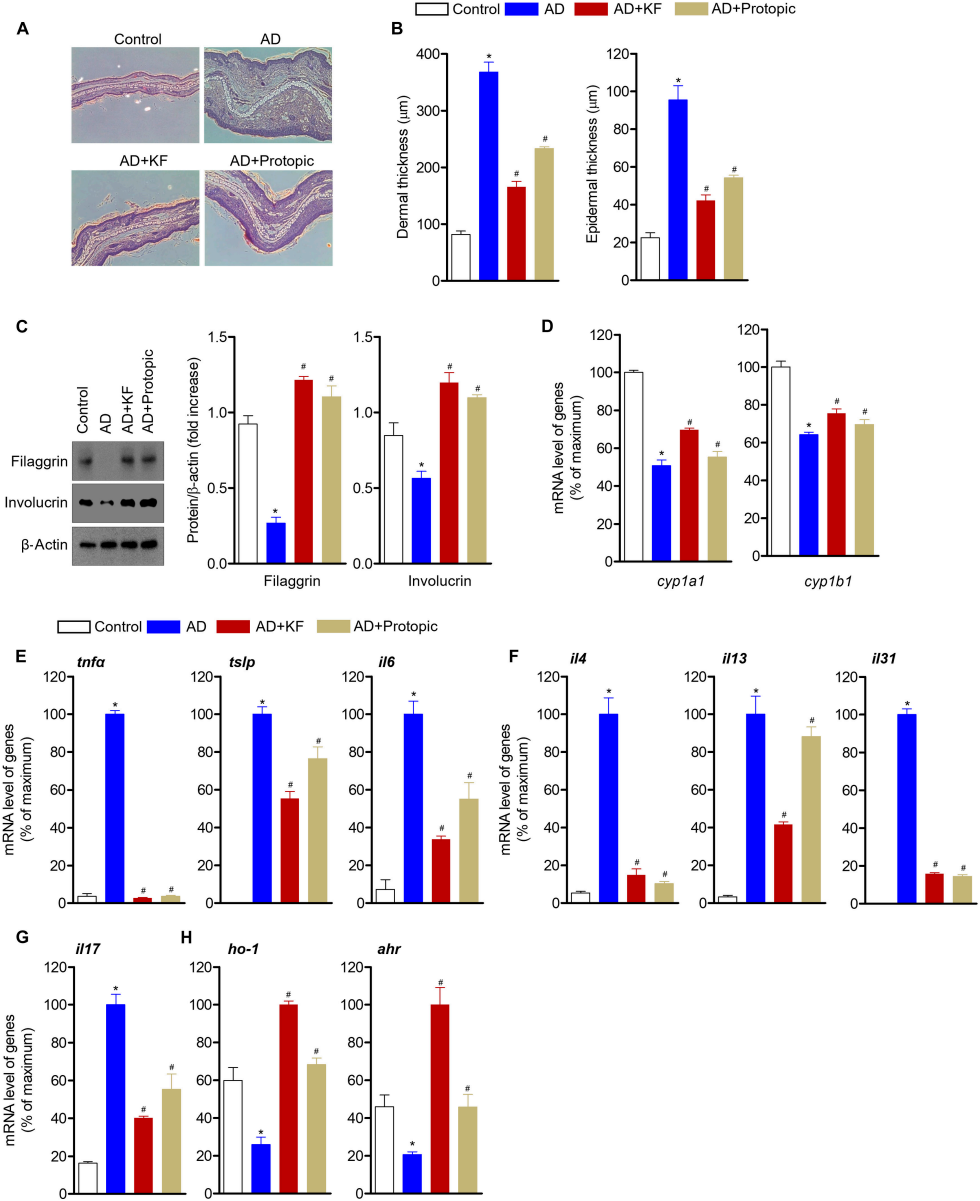
KF modulates the expression of inflammatory cytokines and HO-1 through AhR regulation along with the promotion of epidermal terminal differentiation of ear tissue. (A and B) Hematoxylin and eosin (H&E) images of ears from each group and thickness of dermis and epidermis from the H&E images of ears. (C and D) mRNA levels of the indicated genes or indicated protein levels on ear tissues were evaluated using western blotting and qPCR. (E to H) The mRNA levels of the indicated genes (*tnfα*, *tslp*, *il6*, *il4*, *il13*, *il31*, *il17*, *ho-1*, and *ahr*) on ear tissues were evaluated using qPCR. All gene expressions were normalized to *Gapdh* expression. Results are expressed as the mean ± SEM of 5 mice (*n* = 5). *P < 0.05 versus the control group. ^#^*P* < 0.05 versus the AD-induced group.

To evaluate microscopic changes following oral administration KF treatment, ear tissues were sectioned and stained with H&E. Acanthosis, parakeratosis, and hyperkeratosis were observed in AD tissues, but a dramatic improvement was observed in AD mice that were orally administered KF (Fig. 8A). Moreover, dermal and epidermal thicknesses were reduced in AD mice treated with oral administration of KF (quantifying recovery efficiency: dermal thicknesses, 70%; epidermal thicknesses, 72%) (Fig. 8B). Because AD skin is characterized by epidermal abnormalities, including terminal differentiation of keratinocytes or accumulation of granulocytes, the thickness of the cornified layer in the epidermis and number of granulocytes in the granular layers were investigated to determine whether the oral administration of KF improves AD manifestation. The terminal differentiation of the epidermis in AD mice after the oral administration of KF progressed further than that in control mice (quantifying recovery efficiency: filaggrin, 144%; involucrin, 223%) (Fig. 8C). The thickness of the cornified layer in ear tissues from AD mice orally administered with KF was improved compared with that in control mice. The number of granulocytes in granular layers was also assessed. To explore whether the KF-mediated enhancement of AhR activity occurs *in vivo*, the mRNA levels of *cyp1a1* and *cyp1b1* were detected in the ear tissue. AhR activity was elevated in mice that were administered KF during AD progression (quantifying recovery efficiency: *cyp1a1*, 38%; *cyp1b1*, 31%) (Fig. 8D). Also, in a DNCB/mite-induced AD model, the expression of pro-inflammatory cytokines produced by keratinocytes, dendritic cells, and T cells was investigated and shown to play a crucial role in the development of AD [38]. To check whether oral administration treatment with KF has inhibitory effects on pro-inflammatory cytokines, ear tissues were extracted, and the mRNA levels of cytokines were measured by quantitative PCR. The levels of pro-inflammatory cytokines including *tnfα*, *tslp*, and *il6*, mainly produced by keratinocytes, were significantly decreased in AD mice treated with oral administration of KF (quantifying recovery efficiency: *tnfα*, 101%; *tslp*, 44%; *il6*, 71%) (Fig. 8E). In AD mice orally administered with KF, down-regulated mRNA expression of Th2-mediated cytokines including *il4*, *il13*, and *il31* was observed (quantifying recovery efficiency: *il4*, 90%; *il13*, 60%; *il31*, 84%) (Fig. 8F). Moreover, the expression of IL-17, a pathogenic cytokine that exacerbates inflammatory diseases, was also decreased (quantifying recovery efficiency: *il17*, 71%) (Fig. 8G). These quantitative PCR results suggest that the oral administration of KF not only locally suppresses pro-inflammatory cytokines generated by keratinocytes but also systemically blocks Th2 and Th17 cytokines released mainly from T lymphocytes. To confirm that the pro-inflammatory cytokine regulatory effect of KF was due to the regulation of AhR and HO-1 expression, mRNA expression was analyzed. KF up-regulated the expression levels of *ahr* and *ho-1*, which were down-regulated by AD induction (quantifying recovery efficiency: *ho-1*, 217%; *ahr*, 314%) (Fig. 8H). These results suggest that oral administration of KF to AD mice not only reduces the thickness of the dermis and epidermis but also increases epidermal terminal differentiation by up-regulating AhR activity.

### Alleviation effect of KF on AD in the RHS model

**Fig. 9.**
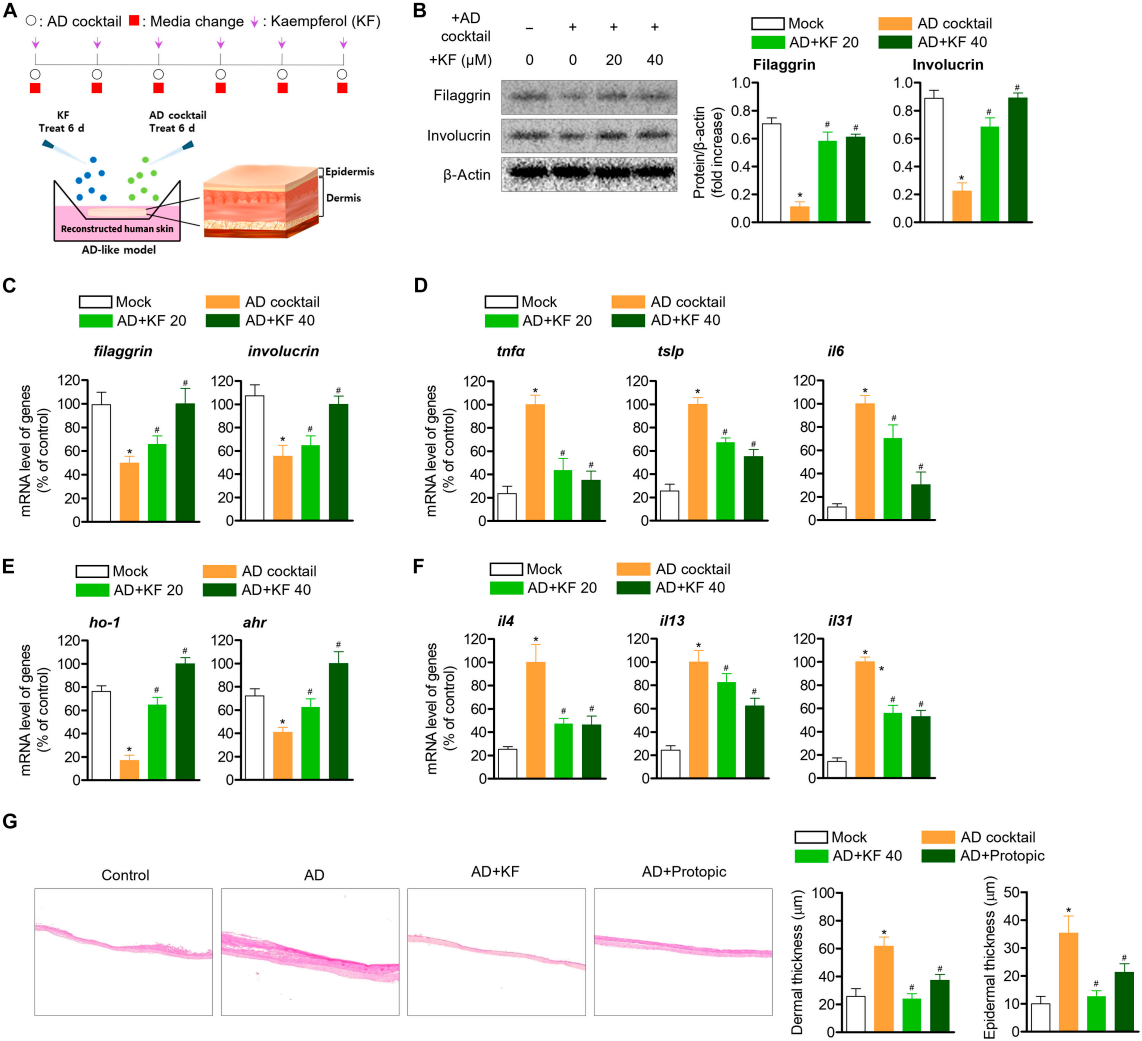
KF reduces skin inflammation in the AD-like reconstructed human skin (RHS) model. (A to F) RHS model inserts were cultured for AD cocktail for 6 d and simultaneously treated with KF (40 μM). After the experiment, proteins and complementary DNA (cDNA) were harvested from the obtained RHS models and western blot and qPCR analyses were performed. In addition, (G) H&E staining was performed to confirm the histological changes of AD-RHS in response to KF. Results are expressed as the mean ± SEM of 5 mice (*n* = 5). *P < 0.05 versus the control group. ^#^*P* < 0.05 versus the AD-induced group.

Finally, to confirm the evidence and effect of KF on AD regulation shown above, we explored its AD mitigation effect in the RHS model. The experimental schedule and method are shown in Fig. 9A. The KF restored epidermal differentiation proteins such as filaggrin and involucrin that were lost by AD cocktail treatment in the RHS model (quantifying recovery efficiency, KF 20 and 40 μM: filaggrin, 79% and 83%; involucrin 69% and 100%) (Fig. 9B) and showed similar results at the gene level (quantifying recovery efficiency, KF 20 and 40 μM: *filaggrin*, 32% and 101%; *involucrin*, 18% and 85%) (Fig. 9C). In addition, it was found that the levels of AD-related cytokines *tnfα*, *tslp*, and *il6* increased by AD cocktail were dose-dependently down-regulated (quantifying recovery efficiency, KF 20 and 40 μM: *tnfα*, 32% and 101%; *tslp*, 18% and 85%; *il6*, 33% and 78%) (Fig. 9D), and the lost *ho-1* and *ahr* gene levels were restored (quantifying recovery efficiency, KF 20 and 40 μM: ho-1, 80% and 139%; ahr, 68% and 188%) (Fig. 9E). These results suggest that KF can exhibit a down-regulating effect on IL produced through Th2 cell polarization by AD, and it was confirmed that KF down-regulated the gene levels of *il4*, *il13*, and *il31* that were up-regulated by AD cocktail in the RHS model (quantifying recovery efficiency, KF 20 and 40 μM: *il4*, 70% and 71%; *il13*, 22% and 49%; *il31*, 51% and 55%) (Fig. 9F). In addition, in the actual RHS model, KF was able to confirm the result of alleviating the thickness of the epidermis and dermis layer thickened by AD induction (quantifying recovery efficiency, KF 20 and 40 μM: dermal thickness, 25% and 104%; epidermis thickness, 10% and 89%) (Fig. 9G), which can be seen as strong evidence for the AD regulation effect of KF through AhR shown previously.

## Discussion

Various *in silico* research techniques are already being used in many pharmaceutical research fields to search for compounds, diseases, and targets, and extensive databases are being built for this purpose [9–12]. However, various treatment methods have previously been proposed for AD, such as controlling the immune response through the inhibition of IL-2 activity, but research on treatment methods that can ultimately link compounds, diseases, and targets is still insufficient. From the research results, we derived overlapping targets by overlapping the relationship between KF’s action targets and AD targets, Afterward, AhR, an interactive gene/protein, was explored as a major candidate for the anti-AD effect through compound–gene target network analysis. In addition, through molecular docking simulations with KF for the retrieved AhR, it was predicted that KF and AhR would exhibit mutual activity at the active site. Therefore, through these predictions, we verified the correlation and pharmacological effects between KF and AhR in AD *in vitro* and *in vivo*.

In the skin, the epidermis plays a critical role by providing a physical but permeable barrier that is continuously reconstructed from the basal layer by the terminal differentiation of the epidermis in a process known as keratinization. Terminal epidermal differentiation involves several steps, from keratinocyte migration from the basal layer to cornification of the outermost skin barrier [7]. The epidermis constitutes only 2.5% of the skin thickness; however, its terminal differentiation imparts protection against invading allergens and pathogens. The expression of proteins such as filaggrin and involucrin is mediated by the complete turnover of the epidermis, including proliferation, differentiation, and death [39]. Filaggrin is one of the most critical proteins involved in terminal epidermal differentiation, and a study on filaggrin-deficient mice reported increased epicutaneous sensitization and skin inflammation [40]. In the present study, we showed elevated cornified layer thickness and restored filaggrin and involucrin expression in the ear tissues of AD mice receiving oral KF administration. The *in vitro* results from HaCaT cells revealed that KF restored the expression of these genes. These results indicate that KF has therapeutic potential for attenuating skin inflammation by up-regulating cornification as a first-line defense mechanism.

Dietary flavonoids have been investigated for decades as therapeutic candidates for multiple diseases owing to their anti-inflammatory and antioxidant effects [41]. Several flavonoids have been reported to suppress AhR transformation in the presence of dioxins, and AhR is considered a useful target for hydroxyflavonoids. These compounds include apigenin, luteolin, galangin, and quercetin [41]. It has now been established that the anti-inflammatory effects of flavonoids occur through ligand binding to AhR [18] Furthermore, induction of the AhR pathway by flavonoids has been shown to trigger the Nrf2 pathway with respect to its cytoprotective effects [42]. These factors promote cross talk between AhR and the Nrf2 pathway via flavonoids, which act as pivotal modulators of skin diseases, including psoriasis and AD [43]. Therefore, several attempts have been made to develop therapeutics for skin inflammation by targeting the AhR and Nrf2 pathways; however, little is known about which bioactive flavonoids are beneficial. Although KF is an AhR ligand, few studies have been conducted to determine whether it exerts its anti-inflammatory effects via the AhR and Nrf2 pathways in skin diseases. The current study suggests that KF exerts therapeutic effects on skin disorders by increasing AhR and Nrf2 activity, as observed in HaCaT cells and an AD mouse model. Our data revealed that treatment with KF enhanced the mRNA levels of Cyp1a1, Cyp1b1, ahr, and epidermal differentiation proteins and decreased the mRNA levels of pro-inflammatory cytokines in HaCaT cells.

SR1 is a small-molecule AhR antagonist originally developed to enhance the self-renewal capacity of human hematopoietic stem cells by inhibiting AhR-mediated differentiation, and due to its relatively high affinity for AhR, SR1 has since been studied in broader contexts, including cancer metastasis, immune modulation, and barrier restoration in epithelial tissues [44]. Compared to other known AhR antagonists such as α-naphthoflavone or indole-3-carbinol metabolite, SR1 exhibits more potent and selective inhibitory activity against AhR [45]. Moreover, several studies have explored whether KF is a potential ligand of AhR in HaCaT cells; however, few studies have compared the affinities of KF and AhR antagonists for AhR [34]. In the present study, SR1 was used as an inhibitor of AhR, and cotreatment with SR1 and KF significantly down-regulated AhR induction. Pretreatment with SR1 did not antagonize the inhibitory effects of KF on the production of pro-inflammatory cytokines.

Previous reports have shown that substances such as medicinal coal tar and soybean tar glyteer have therapeutic effects on inflammatory dermatitis such as AD and psoriasis through regulating oxidative stress by activating AhR [46,47]. However, there have been no published reports on how these natural products affect AhR during the AD process. In this study, AhR was identified as a related target between KF and AD through *in silico* target prediction, and it was also confirmed that KF exerts an alleviating effect on AD by activating Nrf2 as an AD control target. Simultaneously, the disease-alleviating effect of KF through AhR regulation was verified *in vitro* and *vivo* and RHS AD-induced models. In this process, the drug similarity of KF was explored and ADMET prediction was performed to confirm the superiority of KF as a drug, and it was confirmed that intestinal absorption through oral administration of chemically stable KF can show the maximum drug effect. Therefore, KF, as an AD regulator, may serve as a potent orally active inducer of AhR signaling derived from natural sources. In addition, by exploring the targets related to KF and AD through *in silico* analysis and proving the pharmacological efficacy of these, we were able to confirm the new physiological activity of KF, and we aim to suggest insightful results for the development of a new platform that can link natural products and targets and apply them to diseases.

However, the clinical applicability of the study results, which provide more robust evidence for the potential AD therapeutic effect of KF, has several limitations. First, the experimental systems used, including HaCaT keratinocytes and a mouse AD model, provide valuable insights but do not perfectly reproduce the complex human skin or immune responses of AD patients [48]. Furthermore, while KF demonstrated pharmacological efficacy in mice, pharmacokinetic data in humans are limited. The bioavailability, tissue distribution, and metabolic fate of KF and its conjugates in humans are not yet fully understood, and even with allometric scaling, the dose used in mice may not directly correlate with the safe and effective dose in human subjects [49]. Future studies should address these limitations, including primary human keratinocytes, 3D skin models derived from patient samples, pharmacokinetic studies, and early clinical evaluations.

In conclusion, this study proposes the concept that KF functions as an AhR modulator in the skin, highlighting its dual functions of skin barrier restoration and inflammation suppression. In the process, we identify AhR as a novel molecular target of KF and demonstrate that KF directly binds to AhR and activates the AhR–Nrf2 axis, leading to antioxidant effects and restoration of epidermal differentiation proteins. These findings not only reveal novel biological activities of KF but also provide a conceptual framework for the development of natural AhR modulators as promising therapeutics for AD and related skin diseases. Furthermore, by combining *in silico* predictions, mechanistic experiments, and bioavailability and safety profile studies, we present a scalable, integrated research platform that not only accelerates drug target discovery but also suggests promising therapeutic strategies targeting the KF–AhR axis in complex skin pathophysiology at the systems level.

## Data Availability

Data will be made available on request.
