# Supplementary material for: In Silico Target Discovery of Kaempferol: Therapeutic Effect of Kaempferol on Atopic Dermatitis through Regulation of Aryl Hydrocarbon Receptor
Source: Biomater Res. 2025 Nov 11;29:0270. doi: 10.34133/bmr.0270 (PMC12604056; doi:10.34133/bmr.0270)
Supplement: Supplementary 1 — Figs. S1 to S3 Tables S1 to S8 [file bmr.0270.f1.docx]

**Supplementary Materials**

**Title**

Target discovery of kaempferol through in silico prediction:Therapeutic effect of kaempferol on atopic dermatitis through regulation of AhR activation

**Authors**

Eun-Nam Kim^1^,†, Hyun-Su Lee^2^,†, Nguyen Minh Trang1, Jin Tae Hong^3^, Gil-Saeng Jeong^1^,*

**Affiliations**

^1^ College of Pharmacy, Chungnam National University, Daejeon 34134, Republic of Korea; enkim@cnu.ac.kr (E-N. K); ngminhtrang52@gmail.com (M-N. T); gsjeong@cnu.ac.kr (G-S. J)

^2^ Department of Physiology, Daegu Catholic University School of Medicine, Daegu 42472, Republic of Korea; lhs6858@cu.ac.kr (H-S. L)

^3^ College of Pharmacy & Medical Research Center, Chungbuk National University, Cheongju 28160, Republic of Korea; jinthong@chungbuk.ac.kr (J-T. H)

*Correspondence: Professor Gil-Saeng Jeong, College of Pharmacy, Chungnam National University, Daejeon 34134, Republic of Korea. E-mail: gsjeong@cnu.ac.kr

**† These authors share first authorship**

**Contents**

**Table S1.** Primer sequence.

**Table S2.** Overlapping results of KF and AD target.

**Table S3.** Top 20 in STRING network ranked by degree method.

**Table S4.** Top five overlapped targets with a probability of 1.0.

**Table S5.** Binding energies and protein-ligand interaction of KF and potential targets.

**Table S6.** Binding energies and protein-ligand interaction of KF and tapinarof with AhR protein (PDB ID: 7VNA).

**Table S7.** Drug-likeness prediction of KF.

**Table S8.** Identification ADMET of KF

**Fig. S1.** KF and AhR interact by increasing the protein's thermal stability.

**Fig. S2.** AhR silencing reverses KF's regulatory effects on HO-1 and epidermal terminal differentiation proteins.

**Fig. S3.** AhR silencing reverses the anti-inflammatory effects of KF.

**Table S1.** Primer sequence.

| **Target gene** | **Sequence (5′→3′)** | | **Accession Number** |
| --- | --- | --- | --- |
| *CYP1A1* | Forward | GATTGAGCACTGTCAGGAGAA GC | NM_000499 |
|  | Reverse | ATGAGGCTCCAGGAG ATAGCAG |  |
| *CYP1B1* | Forward | GCCACTATCACTGACATCTTCGG | NM_000104 |
|  | Reverse | CACGACCTGATCCAATTCTGCC |  |
| *HO-1* | Forward | CCAGGCAGAGAATGCTGAGTTC | NM_002133 |
|  | Reverse | AAGACTGGGCTCTCCTTGTTGC |  |
| *NQO1* | Forward | CCTGCCATTCTGAAAGGCTGGT | NM_000903 |
|  | Reverse | GTGGTGATGGAAAGCACTGCCT |  |
| *TNFA* | Forward | CTCTTCTGCCTGCTGCACTTTG | NM_000594 |
|  | Reverse | ATGGGCTACAGGCTTGTCACTC |  |
| *TSLP* | Forward | TAGCAATCGGCCACATTGCCT | NM_033035 |
|  | Reverse | GAAGCGACGCCACAATCCTTG |  |
| *IL6* | Forward | AGACAGCCACTCACCTCTTCAG | NM_000600 |
|  | Reverse | TTCTGCCAGTGCCTCTTTGCTG |  |
| *FLG* (filaggrin) | Forward | GCTGAAGGAACTTCTGGAAAAGG | NM_002016 |
|  | Reverse | GTTGTGGTCTATATCCAAGTGATC |  |
| *IVL* (involucrin) | Forward | GGTCCAAGACATTCAACCAGCC | NM_005547 |
|  | Reverse | TCTGGACACTGCGGGTGGTTAT |  |
| *GAPDH* | Forward | GTCTCCTCTGACTTCAACAGCG | NM_002046 |
|  | Reverse | ACCACCCTGTTGCTGTAGCCAA |  |
| *Cyp1a1* | Forward | CATCACAGACAGCCTCATTGAGC | NM_001136059 |
|  | Reverse | CTCCACGAGATAGCAGTTGTGAC |  |
| *Cyp1b1* | Forward | GCCACTATTACGGACATCTTCGG | NM_009994 |
|  | Reverse | ACAACCTGGTCCAACTCAGCCT |  |
| *Ho-1* | Forward | CACTCTGGAGATGACACCTGAG | NM_010442 |
|  | Reverse | GTGTTCCTCTGTCAGCATCACC |  |
| *Noq1* | Forward | GCCGAACACAAGAAGCTGGAAG | NM_008706 |
|  | Reverse | GGCAAATCCTGCTACGAGCACT |  |
| *Tnfa* | Forward | GGTGCCTATGTCTCAGCCTCTT | NM_013693 |
|  | Reverse | GCCATAGAACTGATGAGAGGGAG |  |
| *Tslp* | Forward | GCAAATCGAGGACTGTGAGAGC | NM_021367 |
|  | Reverse | TGAGGGCTTCTCTTGTTCTCCG |  |
| *Il6* | Forward | TACCACTTCACAAGTCGGAGGC | NM_031168 |
|  | Reverse | CTGCAAGTGCATCATCGTTGTTC |  |
| *Il4* | Forward | ATCATCGGCATTTTGAACGAGGTC | NM_021283 |
|  | Reverse | ACCTTGGAAGCCCTACAGACGA |  |
| *Il13* | Forward | AACGGCAGCATGGTATGGAGTG | NM_008355 |
|  | Reverse | TGGGTCCTGTAGATGGCATTGC |  |
| *Il31* | Forward | CAGGTGTCCACTCCCAGGTCCAAG | NM_029594.1 |
|  | Reverse | GGCAACTAGAAGGCACAGTCGAGG |  |
| *Il17* | Forward | CAGACTACCTCAACCGTTCCAC | NM_010552 |
|  | Reverse | TCCAGCTTTCCCTCCGCATTGA |  |
| *Ahr* | Forward | TAATATACCTGCTTCTGGCCGT | NM_013464.2 |
|  | Reverse | GCAGCAAAGGGTGTATCACATA |  |
| *Gapdh* | Forward | CATCACTGCCACCCAGAAGACTG | NM_008084 |
|  | Reverse | ATGCCAGTGAGCTTCCCGTTCAG |  |

**Table S2.** Overlapping results of KF and AD target.

| **No.** | **Target** | **Common name** |
| --- | --- | --- |
| 1 | Carbonic anhydrase II | CA2 |
| 2 | Arachidonate 5-lipoxygenase | ALOX5 |
| 3 | Aryl hydrocarbon receptor | AHR |
| 4 | Carbonic anhydrase XII | CA12 |
| 5 | P-glycoprotein 1 | ABCB1 |
| 6 | ATP-binding cassette sub-family G member 2 | ABCG2 |
| 7 | Adenosine A1 receptor | ADORA1 |
| 8 | Monoamine oxidase A | MAOA |
| 9 | Glyoxalase I | GLO1 |
| 10 | Tyrosine-protein kinase SYK | SYK |
| 11 | Glycogen synthase kinase-3 beta | GSK3B |
| 12 | Matrix metalloproteinase 9 | MMP9 |
| 13 | Matrix metalloproteinase 2 | MMP2 |
| 14 | Arachidonate 15-lipoxygenase | ALOX15 |
| 15 | Arachidonate 12-lipoxygenase | ALOX12 |
| 16 | Adenosine A2a receptor | ADORA2A |
| 17 | Arginase-1 | ARG1 |
| 18 | Estrogen receptor beta | ESR2 |
| 19 | Transthyretin | TTR |
| 20 | Cytochrome P450 19A1 | CYP19A1 |
| 21 | Epidermal growth factor receptor erbB1 | EGFR |
| 22 | Myeloperoxidase | MPO |
| 23 | Tyrosine-protein kinase SRC | SRC |
| 24 | Focal adhesion kinase 1 | PTK2 |
| 25 | Matrix metalloproteinase 13 | MMP13 |
| 26 | Matrix metalloproteinase 3 | MMP3 |
| 27 | Carbonic anhydrase III | CA3 |
| 28 | Interleukin-8 receptor A | CXCR1 |
| 29 | Serine/threonine-protein kinase AKT | AKT1 |
| 30 | Beta-secretase 1 | BACE1 |
| 31 | Aldo-keto-reductase family 1 member C3 | AKR1C3 |
| 32 | Poly [ADP-ribose] polymerase-1 | PARP1 |
| 33 | Matrix metalloproteinase 12 | MMP12 |
| 34 | Lymphocyte differentiation antigen CD38 | CD38 |
| 35 | Estrogen receptor alpha | ESR1 |
| 36 | Cyclooxygenase-2 | PTGS2 |
| 37 | Cystic fibrosis transmembrane conductance regulator | CFTR |
| 38 | 6-phosphofructo-2-kinase/fructose-2,6-bisphosphatase 3 | PFKFB3 |

**Table S3.** Top 20 in STRING network ranked by degree method.

| No. | Common name | Score |
| --- | --- | --- |
| 1 | CA2 | 26 |
| 2 | ALOX5 | 21 |
| 3 | AHR | 19 |
| 4 | CA12 | 18 |
| 5 | ABCB1 | 17 |
| 6 | ABCG2 | 17 |
| 7 | ADORA1 | 13 |
| 8 | MAOA | 10 |
| 9 | GLO1 | 9 |
| 10 | SYK | 9 |
| 11 | GSK3B | 9 |
| 12 | MMP9 | 9 |
| 13 | MMP2 | 8 |
| 14 | ALOX15 | 8 |
| 15 | ALOX12 | 8 |
| 16 | ADORA2A | 7 |
| 17 | ARG1 | 7 |
| 18 | ESR2 | 6 |
| 19 | TTR | 6 |
| 20 | CYP19A1 | 5 |

**Table S4.** Top five overlapped targets with a probability of 1.0.

| **Target** | **Common name** | **Probability** |
| --- | --- | --- |
| Carbonic anhydrase 2 | CA2 | 1 |
| Arachidonate 5-lipoxygenase | ALOX5 | 1 |
| Aryl hydrocarbon receptor | AHR | 1 |
| Carbonic anhydrase 12 | CA12 | 1 |
| ATP Binding Cassette Subfamily B Member 1 | ABCB1 | 1 |

**Table S5.** Binding energies and protein-ligand interaction of KF and potential targets.

| **PDB ID** | **Binding energy (kcal/mol)** | **Hydrogen bonds** | **Van der Waals interactions** | **Hydrophobic interactions** |
| --- | --- | --- | --- | --- |
| 7VNA | -7.6 | Ala551  Ala606 | Asn180  Gln609  Phe 555  Ser670 | Pro668 (π-akyl) Ala672 (π-akyl) Leu607 (π-akyl) |
| 3O8Y | -7.4 | Ala551  Ala606 | Asn180  Gln609  Phe555  Ser670 | Pro668 (π-akyl)  Leu607 (π-akyl)  Ala672 (π-akyl) |
| 1A42 | -6.3 | Thr200  Thr199  Pro202 |  | Leu198 (π-π stacked)  Phe131 (π-π stacked)  His94 (π-π T-shaped)  Val121 (π-akyl) |
| 1JCZ | -6.3 | Gln92  Ser135  Thr91  Pro201  Pro202  Thr200 |  | Lys67 (π-cation)  His94 (π-π T-shaped)  Ala131 (π-akyl) |
| 7OTI | -4.4 | Thr172 | Thr169  Asp173 | Arg170 (π-akyl) |

**Table S6.** Binding energies and protein-ligand interaction of KF and tapinarof with AhR protein (PDB ID: 7VNA).

| **Compound** | **Binding energy (kcal/mol)** | **Hydrogen bonds** | **Van der Waals interactions** | **Hydrophobic interactions** |
| --- | --- | --- | --- | --- |
| Tapinarof | -7.1 | His366 | - | Tyr336(π-π stacked)  Lys289 (π-akyl) |

**Table S7.** Drug-likeness prediction of KF.

| **Properties** | **KF** | **Level** |
| --- | --- | --- |
| MW | 286.24 | - |
| Fomular | C_15_H_10_O_6_ | - |
| Lipinski's Rule | 0 violation | Good |
| Lead-like Rule | 0 violation | Good |
| Molecular refractivity | 76.01 | Good |
| HBD (H-Bond Donors) | 4 | Good |
| HBA (H-Bond Acceptors) | 6 | Good |
| No. rotatable bonds | 1 | Good |
| Aromatic Rings | 3 | Good |
| logP | 2.12 | Optimal |
| Solubility in pure water (mg/l, pH6.5) | 350.00 | Slightly soluble |

**Table S8.** Identification ADMET of KF.

| **ADME prediction** | | **KF** | | **Level** |
| --- | --- | --- | --- | --- |
| MW | | 286.24 | | - |
| Fomular | | C_15_H_10_O_6_ | | - |
| Human intestinal absorption  (Max. passive Abosrption %) | | 100 %  Transcellular 100% Paracellular route = 0 % | | Highly absorbed |
| BBB  (Brain/Plasma equilibration rate) | | -3.50 | | CNS inactive due to low brain penetration |
| CNS Score | | -4.05 | | No penetrant |
| Plasma Protein Binding (%PPB) | | 93.48  (RI = 0.34) | | Extensively bound |
| **Toxicity Prediction** | | **KF** | **Level** | |
| Acute Tox LD50 (Mouse) | Oral | 1,0000 mg/kg | (RI = 0.33) | |
|  | Intravenous | 490 mg/kg | (RI = 0.66) | |
|  | Subcutaneous | 410 mg/kg | (RI = 0.67) | |
|  | Intraperitoneal | 350 mg/kg | (RI = 0.67) | |
| Acute Tox LD50 (Rat) | Oral | 610 mg/kg | (RI = 0.51) | |
|  | Intraperitoneal | 1,600 mg/kg | (RI = 0.39) | |
| Acute(LD50) Category | | (50~300,~2,000  mg/kg) | Category 3,4 | |
| DNA Damage | | Borderline | Outside Applicable Domain | |
| Carcinogenicity | | Borderline | Negative | |
| Chromosome Aberration | | Borderline | Outside Applicable Domain | |
| Ames Mutagenicity | | Mutagenic | 0.92 (RI = 0.87) | |

**
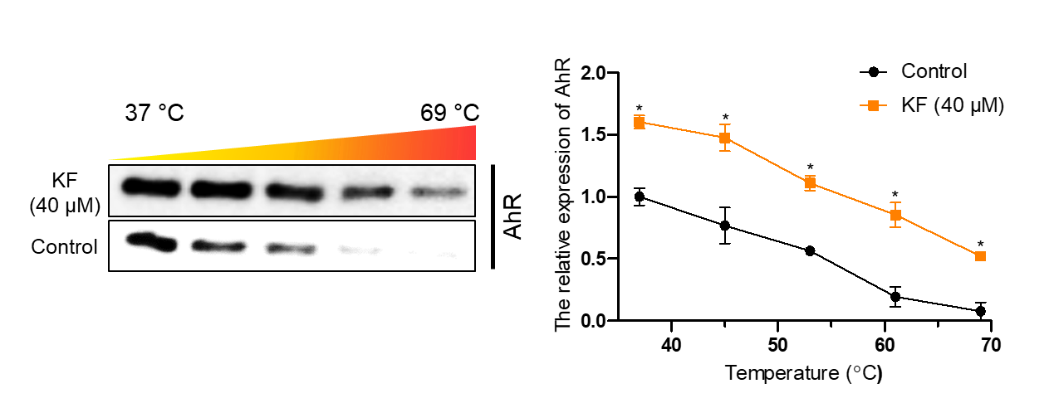
**

**Fig. S1.** KF and AhR interact by increasing the protein's thermal stability. *, *P*<0.05, versus the each temperature control group.

**
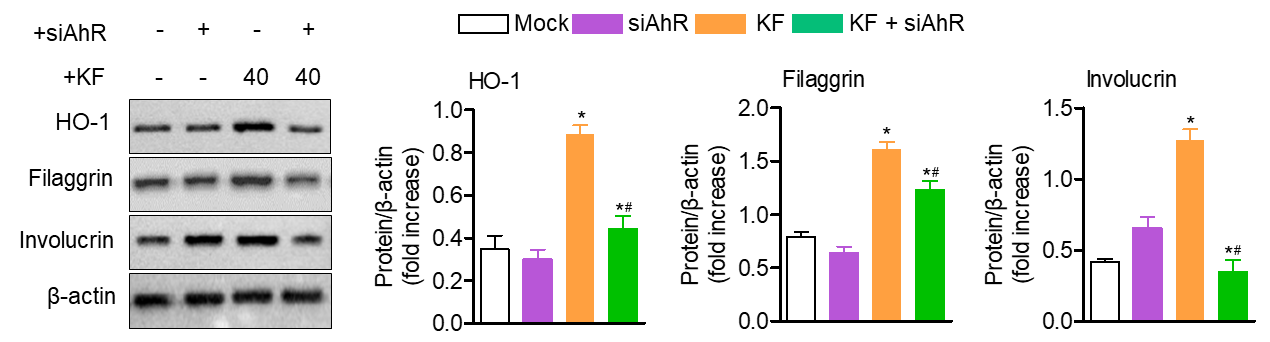
**

**Fig. S2.** AhR silencing reverses KF's regulatory effects on HO-1 and epidermal terminal differentiation proteins. Results are expressed as mean ± SEM (*n*=3). *, *P*<0.05, versus the only siAhR treat group. #, *P*<0.05, versus the KF treat group.

**
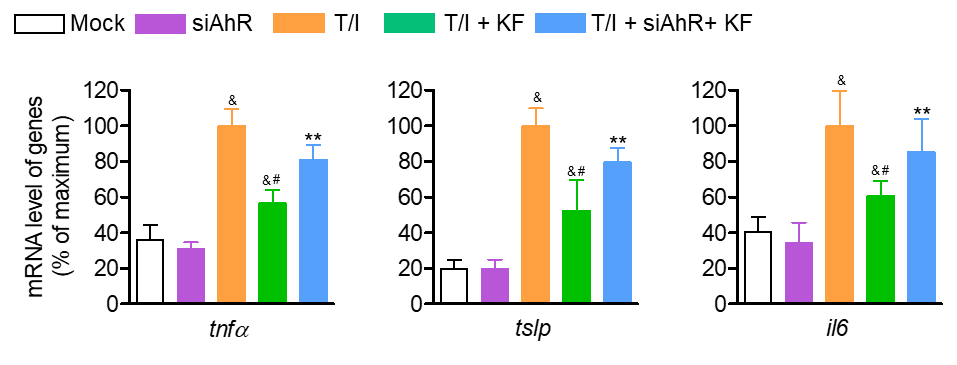
**

**Fig. S3.** AhR silencing reverses the anti-inflammatory effects of KF. Results are expressed as mean ± SEM (*n*=3). #, *P*<0.05, versus the only T/I treat group. &, *P*<0.05, versus the only siAhR treat group. **, *P*<0.05, versus the only T/I+KF treat group.
